# Endothelial cell–specific postnatal deletion of *Nos3* preserves intraocular pressure homeostasis via macrophage recruitment and NOS2 upregulation

**DOI:** 10.1172/JCI183440

**Published:** 2025-02-11

**Authors:** Ruth A. Kelly, Megan S. Kuhn, Ester Reina-Torres, Revathi Balasubramanian, Kristin M. Perkumas, Guorong Li, Takamune Takahashi, Simon W.M. John, Michael H. Elliott, Darryl R. Overby, W. Daniel Stamer

**Affiliations:** 1 Department of Ophthalmology, Duke Eye Center, Duke University, Durham, North Carolina, USA.; 2 Department of Bioengineering, Imperial College London, London, United Kingdom.; 3 Department of Ophthalmology, Columbia University, New York, New York, USA.; 4 Division of Nephrology and Hypertension, Vanderbilt University, Nashville, Tennessee, USA.; 5 Department of Ophthalmology, Dean McGee Eye Institute and; 6 Department of Biochemistry & Physiology, University of Oklahoma Health Sciences Center (OUHSC), Oklahoma City, Oklahoma, USA.

**Keywords:** Immunology, Ophthalmology, Vascular biology, Endothelial cells, Macrophages, Nitric oxide

## Abstract

Polymorphisms in *Nos3* increase risk for glaucoma, the leading cause of irreversible blindness worldwide. A key modifiable risk factor for glaucoma is intraocular pressure (IOP), which is regulated by NO — a product of nitric oxide synthase 3 (encoded by Nos3) — in Schlemm’s canal of the conventional outflow pathway. We studied the effects of a conditional, endothelial cell–specific postnatal deletion of *Nos3* (*Endo-SclCre-ER^T^;Nos3^fl/fl^*) on tissues of the outflow pathway. We observed that Cre-ER^T^ expression spontaneously and gradually increased with time in vascular endothelia including in Schlemm’s canal, beginning at P10, with complete *Nos3* deletion occurring around P90. Whereas outflow resistance was reduced in global *Nos3*-KO mice, outflow resistance and IOP in *Endo-SclCre-ER^T^;Nos3^fl/fl^* mice were normal. We observed — coincident with *Nos3* deletion — recruitment of macrophages to and induction of both ELAM1 and NOS2 expression by endothelia in the distal portion of the outflow pathway, which increased vessel diameter. These adjustments reduced outflow resistance to maintain IOP in these *Endo-SclCre-ER^T^;Nos3^fl/fl^* mice. Selective inhibition of iNOS by 1400W resulted in narrowing of distal vessels and IOP elevation. Together, the results emphasize the pliability of the outflow system and the importance of NO signaling in IOP control, and imply an substantial role for macrophages in IOP homeostasis.

## Abstract 2

This study emphasizes the pliability of the outflow system, the importance of NO signaling on IOP control and implicates an important role for macrophages in IOP homeostasis.

## Graphical abstract

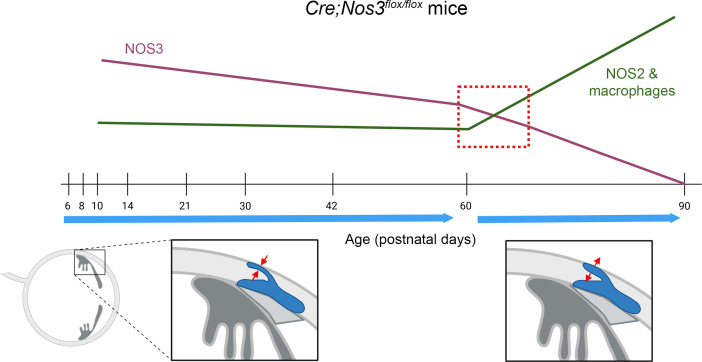

## Introduction

Glaucoma is a complex group of blinding diseases that affects millions of people worldwide, with numbers expected to reach 111.8 million by 2040 (1). Intraocular pressure–lowering (IOP-lowering) medications and a variety of surgical procedures are effective treatments for glaucoma, as IOP remains the only modifiable risk factor (2). IOP homeostasis and dysregulation are controlled by the cells of the conventional outflow pathway, which continually monitor and respond to changes in IOP (3). As IOP increases, the tissues that regulate the resistance to drainage of aqueous humor distend outward (4–8). This results in narrowing of the lumen of Schlemm’s canal (SC), which is a unique circular hybrid lymphatic/vascular drainage vessel (9). Thus, to exit the eye, aqueous humor must pass through a narrower SC lumen, producing increased shear stress, activation of *Nos3*, and production of NO (3, 7, 10–14).

NO produced by SC lowers resistance to aqueous humor outflow, thereby normalizing IOP through several mechanisms, including relaxation of the trabecular meshwork (TM) (14–16), dilation of venous vessels distal to SC (17), and increased permeability of SC’s inner wall (18). In contrast, inhibition of *Nos3* increases resistance to aqueous humor outflow and IOP (10, 14, 19, 20). Aligning with NO’s role as a mechanosensitive regulator of IOP homeostasis, NO production and *Nos3* expression by SC are sensitive to changes in IOP (3, 11, 19). Moreover, transgenic mice overexpressing *Nos3* have lower IOPs and lower outflow resistance, whereas *Nos3*-KO mice have higher IOPs and higher outflow resistance (10, 20, 21). In other words, *Nos3* is involved in transducing IOP-dependent changes into effects that modulate outflow resistance. Importantly, these findings in mice translate to humans, where polymorphisms in the *Nos3* gene associate with increased risk of elevated IOP and glaucoma in genetic linkage studies involving several populations (22–27).

Since global Nos3-KO mice tested previously had germline deletions and were subject to developmental defects (e.g., malformations in heart and lungs are common in global Nos3-KO mice) (28, 29), there was potential for early compensatory changes that may have affected outflow function. We therefore explored the effects of a conditional, endothelial cell–specific postnatal deletion of *Nos3* (*Endo-SclCre-ER^T^;Nos3^fl/fl^*) on outflow morphology, function, and IOP.

## Results

### Spontaneous Cre-ER^T^ recombination observed in 2 independent reporter mouse strains.

**Figure 1:**
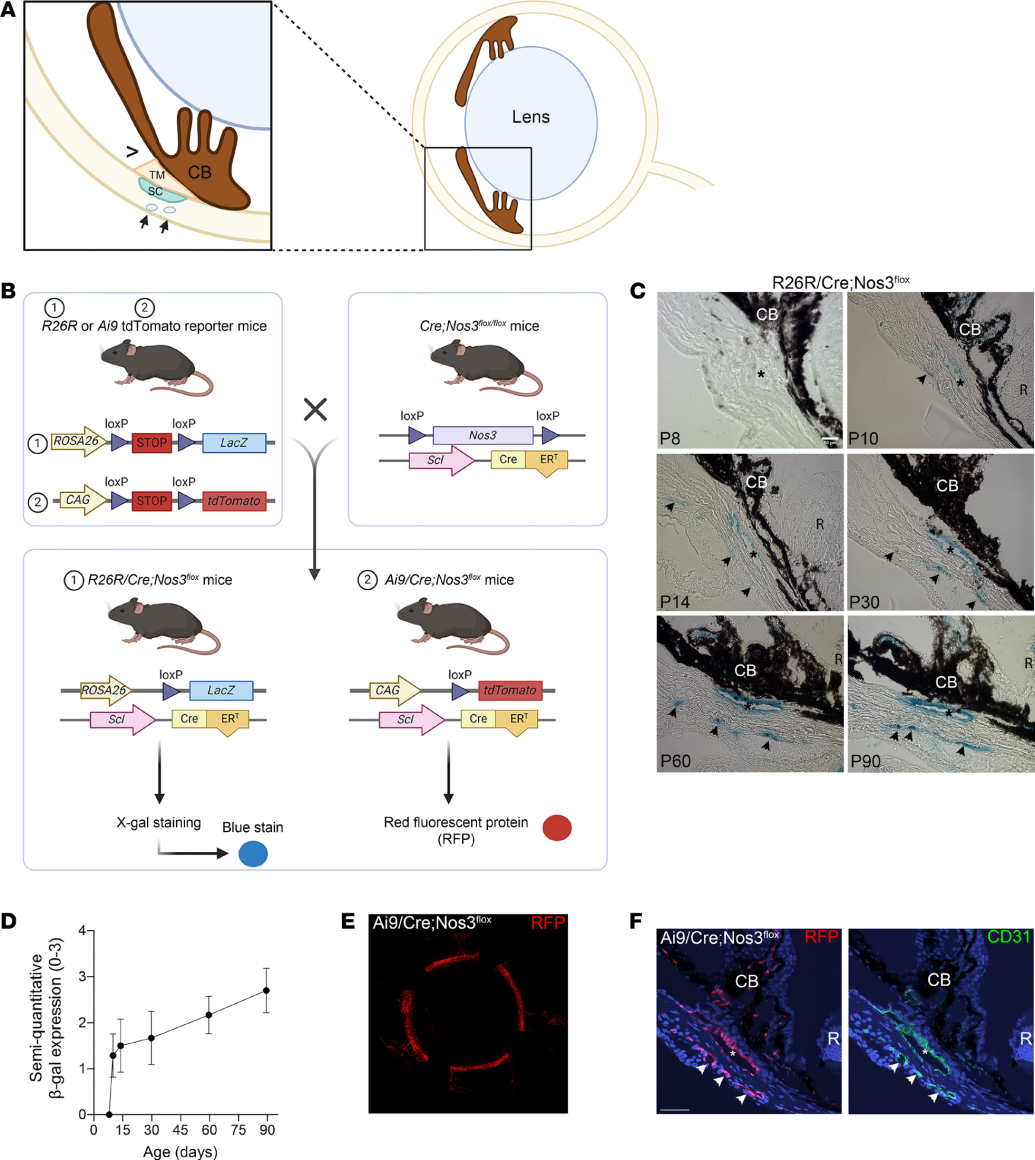
Spontaneous Cre activity in endothelial cells of SC and the DV in 2 different reporter mouse strains. (**A**) Schematic showing localization of tissues of the conventional outflow pathway in cross section of mouse eye. The left panel shows a magnified view of conventional outflow tissues in the right panel (indicated by the box). (**B**) Schematic showing both *R26R* and *Ai9* tdTomato reporter mouse strains crossed with *Cre;Nos3^fl/fl^* mice to give *R26R/Cre;Nos3^fl^* mice and *Ai9/Cre;Nos3^fl^* mice, respectively. X-gal staining was carried out on tissue from *R26R/Cre;Nos3^fl^* mice to identify LacZ expression (blue), indicating Cre activity; and RFP indicates Cre activity in *Ai9/Cre;Nos3^fl^* mice. (**C**) LacZ expression (blue), indicating Cre-ER^T^ activity, was apparent in endothelial cells of SC (*) and the surrounding DV (arrows) in *R26R/Cre;Nos3^fl^* mice. Staining was also present in the CB, which also contains blood vessels. There was no blue stain present in the P8 mice examined. Light blue staining appeared at P10 and intensified with age until P90. Images shown are representative of *n* = 8 eyes, 10–15 sections per eye from each quadrant (from both male and females) from each age group. (**D**) Semiquantitative scoring of X-gal staining, which was carried out by 2 trained observers masked to the experimental protocol, showed increased stain intensity with age. (**E**) Flat-mount image showing RFP (red) expression along the entire SC and DV of *Ai9/Cre;Nos3^fl^* mice at P30. Image represents *n* = 6 eyes (from both males and females). (**F**) Cross-section images showing RFP (red) in endothelial cells of the SC and the DV, following the same pattern as the endothelial cell marker CD31 (green). Images represent *n* = 8 eyes, 10–15 sections per eye from each quadrant (from both male and females). Scale bars: 50 μm in all images. R, retina. Asterisk: SC; arrows: DV.

The *Endo-SclCre-ER^T^;Nos3^fl/fl^* mice used throughout this study are referred to herein as *Cre;Nos3^fl/fl^*. The location of the conventional outflow pathway of the mouse eye is shown schematically in cross-section (Figure 1A). Cre-ER^T^ activity in tissue sections of the conventional outflow pathway of *R26R* and *Ai9* reporter mice crossed with *Cre;Nos3^fl/fl^* mice (producing *R26R/Cre;Nos3^fl^* and *Ai9/Cre;Nos3^fl^* mice, respectively) was localized using LacZ expression/X-gal cleavage and red fluorescent protein (RFP) (Figure 1B). In pilot studies, LacZ expression was unexpectedly present in endothelial cells of SC, the surrounding distal vasculature (DV) (arrows), ciliary body (CB), and iris ocular tissues of all *R26R/Cre;Nos3^fl^* mice at P60–P90 (*n* = 12 eyes), whether or not they had received topical tamoxifen treatment (Supplemental Figure 1; supplemental material available online with this article; https://doi.org/10.1172/JCI183440DS1). Topical administration of tamoxifen is an established and effective route for local targeting of ocular tissues, with limited off-target systemic effects (30, 31). As a noninvasive method of delivery, it also greatly reduces the potential risk of infections or toxicity due to systemic routes of injections (32). Due to the spontaneous Cre-ER^T^ recombination observed in *R26R/Cre;Nos3^fl^* mice, we sought to identify at what age this Cre-ER^T^ recombination was occurring. Therefore, X-gal staining was carried out on ocular tissue from *R26R/Cre;Nos3^fl^* mice at P8, P10, P14, P30, P60, and P90 without tamoxifen treatment. While X-gal staining was absent in all mice examined at P8, light staining of SC, the surrounding DV, ciliary body, and iris tissue was apparent at P10. This staining intensified with the age of the animal, until reaching a dark blue stain at P90 (*n* = 8 eyes, 10–15 sections per eye; Figure 1C). To record the intensity change in X-gal staining with age among all mice examined, 2 trained observers masked to the experimental protocol scored the staining in a semiquantitative fashion). The images were scored based on a scoring system from 0 to 3 (no stain = 0, very light staining = 1, darker staining = 2, and very intense staining = 3) (Figure 1D). Each observer was given an example image to represent each score. The results shown are an average of the 2 observers’ scores for each time point shown in Figure 1C. To ensure the robustness of this approach, inter-rater reliability (κ coefficient) was calculated for both trained observers (33) to be 0.76, which represents “substantial agreement” between observers. LacZ staining results were confirmed in a second reporter line (*Ai9*) (Figure 1, E and F).

### Nos3/NOS3 deletion occurs gradually over time in Cre;Nos3^fl/fl^ mice.

**Figure 2:**
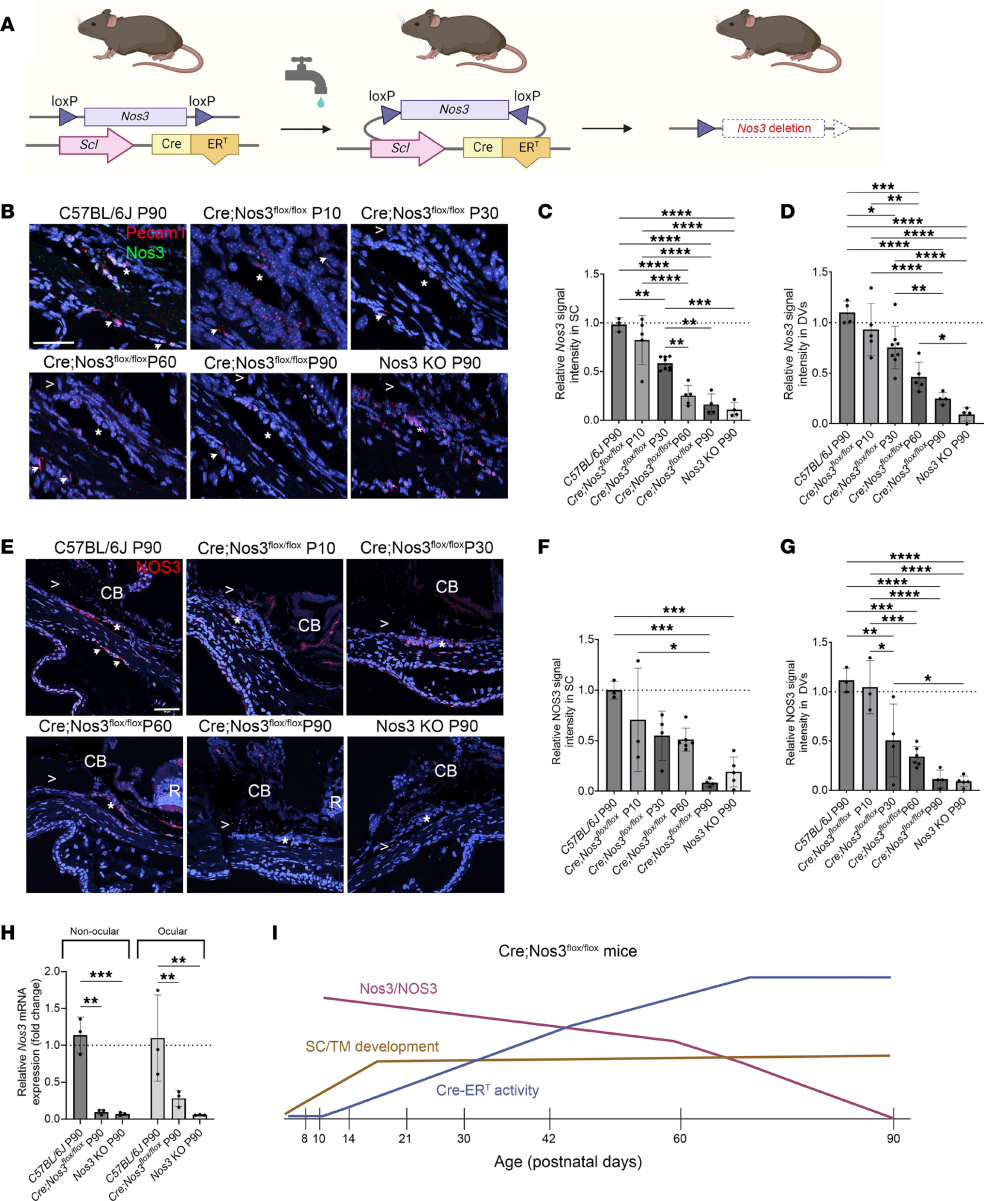
Reduced *Nos3*/NOS3 expression was observed in SC and distal vessels with age in *Cre;Nos3^fl/fl^* mice. (**A**) Schematic showing *Nos3* deletion in the absence of tamoxifen due to a leaky promoter (indicated by faucet). (**B**) Representative images of RNAscope labeling at each age are shown. Staining for nuclei (DAPI) is in blue. >: open iridocorneal angle; arrows: DV; asterisks: SC. Images are representative of *n* = 3–6 eyes, 5–10 sections per eye for each time point. (**C**) A significant decrease in Nos3 signal intensity in SC was observed in Cre;Nos3fl/fl mice compared to C57BL/6J mice from P10 to P90 (*P* < 0.0001). Each data point represents a single unpaired eye. *P < 0.05, **P < 0.01, ***P < 0.001, and ****P < 0.0001; 1-way ANOVA with Tukey’s multiple-comparison test. (**D**) Significant loss of *Nos3* in DVs paralleled that in SC in *Cre;Nos3^fl/fl^* mice by P90 compared with C57BL/6J mice at P90 (*P* < 0.0001). This loss of *Nos3* in *Cre;Nos3^fl/fl^* mice at P90 was no different from in *Nos3*-KO mice at P90 (*P* > 0.9999). (**E**) Representative images of NOS3 expression at each age. (**F**) Relative NOS3 signal intensity in SC of *Cre;Nos3^fl/fl^* mice showed a steady loss of NOS3 in SC from P10 to P90, with a significant difference from C57BL/6J mice at P90 (*P* = 0.0003) but no difference from *Nos3*-KO mice at P90 (*P* = 0.9943). (**G**) NOS3 signal intensity with age in the DVs looked like the SC region. (**H**) Real-time PCR of eye and lung showed *Nos3* RNA expression in *Cre;Nos3^fl/fl^* mice resembling that in *Nos3*-KO mice, compared with C57BL/6J mice, at P90. (**I**) Schematic showing the expression patterns of *Nos3* mRNA, NOS3 protein, SC/TM development, and Cre-ER^T^ activity with age in *Cre;Nos3^fl/fl^* mice.

We next determined whether increased Cre-ER^T^ activity with age corresponded to decreased *Nos3* expression in *Cre;Nos3^fl/fl^* mice in the absence of tamoxifen treatment (Figure 2A). We examined *Nos3* mRNA and NOS3 protein using RNAscope and IHC, respectively. RNAscope (ACD Bio) has become a standard ISH technique. Its sensitivity and specificity allow for detection of low-abundance mRNAs in single cells and in a short amount of time (34). Both techniques were carried out on fixed-frozen ocular cryosections that contained the conventional outflow pathway from *Cre;Nos3^fl/fl^* mice aged P10 to P90. For comparison, we examined C57BL/6J control mice and *Nos3*-KO mice, both at P90. Representative images of RNAscope labeling at each age are shown. Probes for *Pecam-1* (red) and *Nos3* (green) were used to identify endothelial cells and relative mRNA expression of *Nos3*, respectively. DAPI (blue) was used to label cell nuclei (Figure 2B). To quantify expression, we counted fluorescent puncta corresponding to each individual *Nos3* mRNA molecule that overlapped with DAPI staining along the SC lumen and DV (Supplemental Figure 2), expressed the level as percentage of puncta per area of SC (circumferential length) or DV region, and normalized the values to expression by WT C57BL/6J mice (Figure 2, C and D). Data points in Figure 2, C and D, are the average of approximately 10 tissue sections of the same eye. A significant decrease in relative *Nos3* signal intensity in the SC region was observed in *Cre;Nos3^fl/fl^* mice at P30 (0.6 ± 0.07, *P* = 0.0086, *n* = 8 eyes), P60 (0.3 ± 0.1, *P* < 0.0001, *n* = 5 eyes), and P90 (0.2 ± 0.1, *P* < 0.0001, *n* = 4 eyes) compared with C57BL/6J mice at P90 (1.0 ± 0.07, *n* = 3 eyes; mean ± SD, 1-way ANOVA with Tukey’s multiple-comparison test; Figure 2C). A significant decrease in relative *Nos3* signal intensity was observed in *Cre;Nos3^fl/fl^* mice at P10 (0.8 ± 0.3, *P* < 0.0001, *n* = 5 eyes) and P30 (0.6 ± 0.1, *P* = 0.0003, *n* = 8 eyes) compared with *Nos3*-KO mice at P90 (0.1 ± 0.07, *n* = 4 eyes). A significant decrease in relative *Nos3* signal intensity in the SC region was also observed in *Cre;Nos3^fl/fl^* mice at P60 (0.3 ± 0.1, *P* = 0.01, *n* = 5 eyes) and P90 (0.2 ± 0.1, *P* = 0.0014, *n* = 4 eyes) compared with *Cre;Nos3^fl/fl^* mice at P30 (0.6 ± 0.07, *n* = 8 eyes). These data suggest that *Nos3* mRNA expression in SC in *Cre;Nos3^fl/fl^* mice deviated from that of C57BL/6J mice around P30 and began to resemble that in *Nos3*-KO mice at P60 and P90. A gradual and significant decrease in *Nos3* expression in SC was observed with age in *Cre;Nos3^fl/fl^* mice so that at P90 *Nos3* expression was no different from that in *Nos3*-KO mice at P90 (0.2 ± 0.1 vs. 0.1 ± 0.07, *P* = 0.9995).

Relative *Nos3* mRNA expression in the DV for *Cre;Nos3^fl/fl^* mice at P90 (0.3 ± 0.06, *n* = 3, Figure 2D) and *Nos3*-KO mice at P90 (0.09 ± 0.07, *n* = 3) was significantly different from that for C57BL/6J mice at P90 (1.1 ± 0.1, *P* < 0.0001 for both, *n* = 4). Supporting total *Nos3* ablation at P90, relative *Nos3* mRNA expression in the DV for *Cre;Nos3^fl/fl^* mice at P90 and *Nos3*-KO mice did not differ (*P* = 0.7926). *Cre;Nos3^fl/fl^* mice at P10 (0.9 ± 0.3, *P* < 0.0001, *n* = 5), P30 (0.8 ± 0.2, *P* < 0.0001, *n* = 8), and P60 (0.5 ± 0.1, *P* = 0.0426, *n* = 5) were significantly different from *Nos3*-KO mice at P90. *Cre;Nos3^fl/fl^* mice at both P10 and P30 were significantly different from *Cre;Nos3^fl/fl^* mice at P90 (*P* < 0.0001 and *P* = 0.001, respectively). *Cre;Nos3^fl/fl^* mice at both P30 and P60 were also significantly different from C57BL/6J mice at P90 (*P* = 0.0346 and *P* = 0.0002, respectively). In addition, *Cre;Nos3^fl/fl^* mice at P10 were significantly different from *Cre;Nos3^fl/fl^* mice at both P60 (*P* = 0.0031) and P90 (*P* < 0.0001).

This pattern of *Nos3* mRNA expression mirrored NOS3 protein levels. Representative images showing NOS3 protein expression at each age are shown in Figure 2E. In WT mice, NOS3 staining was confined to the endothelial cells of SC (*) and surrounding DV (arrows) (Figure 2E). This staining pattern colocalized with CD31 staining, which was used to locate SC and distal endothelia, but was unlike that of α–smooth muscle actin (α-SMA), which was used to identify the TM (Supplemental Figure 3). Consistent with the onset of Cre-ER^T^ activation in *Cre;Nos3^fl/fl^* mice, NOS3 staining gradually decreased in the SC region between the ages of P10 and P60, with little staining appearing at P90. NOS3 staining in both *Cre;Nos3^fl/fl^* mice at P90 (0.1 ± 0.1, *P* = 0.0003, *n* = 4) and *Nos3*-KO mice at P90 (0.2 ± 0.1, *P* = 0.0007, *n* = 5) was significantly different from that in C57BL/6J mice at P90 (1.0 ± 0.1, *n* = 3, Figure 2F). In fact, NOS3 staining in *Cre;Nos3^fl/fl^* mice at P90 was similar to staining in *Nos3*-KO mice at P90 (0.1 ± 0.04 vs. 0.2 ± 0.1, *P* = 0.9943). NOS3 staining in *Cre;Nos3^fl/fl^* mice at P10 (0.7 ± 0.5, *n* = 3) was also significantly different from that in *Cre;Nos3^fl/fl^* mice at P90 (*P* = 0.0181).

Similar to mRNA expression, NOS3 protein expression in the DV for both *Cre;Nos3^fl/fl^* mice at P90 (0.1 ± 0.09, *P* < 0.0001, *n* = 3, Figure 2G) and *Nos3*-KO mice at P90 (0.09 ± 0.05, *P* < 0.0001, *n* = 3) was significantly different from that of C57BL/6J mice at P90 (1.1 ± 0.1, *n* = 3). Relative NOS3 protein expression in the DV of *Cre;Nos3^fl/fl^* mice at P90 and *Nos3*-KO mice at P90 did not differ (*P* > 0.9999). *Cre;Nos3^fl/fl^* mice at both P10 (1.0 ± 0.3, *P* < 0.0001, *n* = 3) and P30 (0.5 ± 0.4, *P* = 0.0387, *n* = 4) were significantly different from *Nos3*-KO mice at P90. *Cre;Nos3^fl/fl^* mice at P10 were significantly different from *Cre;Nos3^fl/fl^* mice at P90 (*P* < 0.0001). *Cre;Nos3^fl/fl^* mice at both P30 and P60 (0.3 ± 0.1, *n* = 6) were also significantly different from C57BL/6J mice at P90 (*P* = 0.0051 and *P* = 0.0002, respectively). Moreover, *Cre;Nos3^fl/fl^* mice at P10 were significantly different from *Cre;Nos3^fl/fl^* mice at P30 (*P* = 0.0141), P60 (*P* = 0.0005), and P90 (*P* < 0.0001).

To examine expression of *Nos3* in *Cre;Nos3^fl/fl^* mice using a third method, we carried out quantitative PCR (qPCR) on both nonocular (lung) and ocular (outflow tissues) tissues from all 3 genotypes at P90. In nonocular tissue, both *Cre;Nos3^fl/fl^* mice (0.1 ± 0.03, *P* = 0.0011, 2-way ANOVA with Tukey’s multiple-comparison test, *n* = 3; Figure 2H) and *Nos3*-KO mice (0.1 ± 0.02, *P* = 0.0009, *n* = 3) had significantly lower levels of *Nos3* mRNA compared with C57BL/6J mice at P90 (1.1 ± 0.2). In ocular tissues, both *Cre;Nos3^fl/fl^* mice (0.3 ± 0.1, *P* = 0.0065, *n* = 3) and *Nos3*-KO mice (0.1 ± 0.0, *P* = 0.0011, *n* = 3) had significantly lower levels of *Nos3* mRNA compared with C57BL/6J mice at P90 (1.1 ± 0.6). There was no difference in *Nos3* mRNA expression between *Nos3*-KO mice at P90 and *Cre;Nos3^fl/fl^* mice at P90 in either nonocular (*P* = 0.9918) and ocular tissues (*P* = 0.5636). A schematic (Figure 2I) compares the expression patterns of *Nos3* mRNA, NOS3 protein, and Cre-ER^T^ activity with age. SC/TM development was included in the schematic as a reference. SC develops over time, beginning from birth, with mature SC cells starting to appear around P10. SC development is largely complete by P17, with minor changes afterward (35). The TM is largely formed by P21, with minor remodeling out to P42 (35).

### In the absence of Nos3 in SC, Cre;Nos3^fl/fl^ mice maintain a healthy outflow facility and IOP.

**Figure 3:**
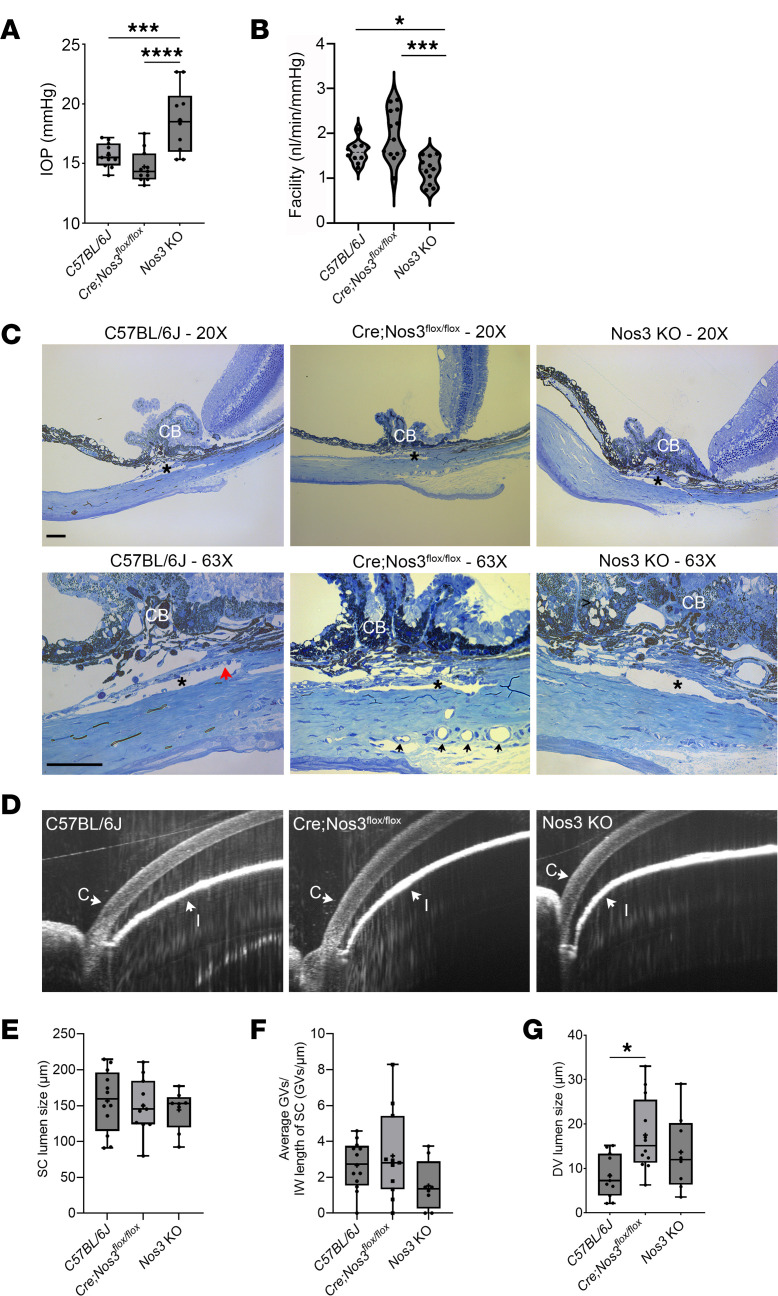
*Cre;Nos3^fl/fl^* mice at P90 have open angles and normal IOPs due to healthy outflow facility, despite loss of *Nos3* in outflow vessels. (**A**) IOP measurements are displayed in box and whisker plots, where the box represents the interquartile range, the horizontal line in the box represents the median, the plus symbol in the box shows the mean, and the whiskers show the minimum and maximum values. Mean IOP measurements of 15.7, 14.7, and 18.6 mmHg were observed in C57BL/6J (*n* = 11 eyes), *Cre;Nos3^fl/fl^* (*n* = 11 eyes), and *Nos3*-KO mice (*n* = 10 eyes), respectively. While IOP of *Cre;Nos3^fl/fl^* mice was not different from that of C57BL/6J mice, *Nos3*-KO mice showed significantly elevated IOP compared with both *Cre;Nos3^fl/fl^* mice (*P* < 0.0001) and C57BL/6J mice (*P* = 0.0027). (**B**) Mean facility values of 1.6, 1.9, and 1.1 nL/min/mmHg were determined in C57BL/6J mice (*n* = 10 eyes), *Cre;Nos3^fl/fl^* mice (*n* = 13 eyes), and *Nos3*-KO mice (*n* = 11 eyes), respectively. *Cre;Nos3^fl/fl^* mice showed a significant increase in outflow facility compared with *Nos3*-KO mice (*P* = 0.0002). (**C**) Representative images of angle structures from methylene blue–stained sections were used to analyze gross morphological differences between strains at P90. Distal vessels (arrows) in *Cre;Nos3^fl/fl^* mice appeared enlarged compared with those in both C57BL/6J and KO mice. The red arrow points to a GV along the inner wall of SC. Asterisk: SC, arrows: DV; and CB: ciliary body. Scale bar: 50 μm. (**D**) Representative OCT images of living mice of each strain at P90 showing open iridocorneal angles (*n* = 2 eyes), with no significant difference in the size of SC lumen (**E**) or the number of GVs per SC (**F**) between strains. *Nos3*-KO mice had fewer GVs per SC compared with both C57BL/6J and *Cre;Nos3^fl/fl^* mice, but not to a significant degree. (**G**) Distal vessels (arrows) in *Cre;Nos3^fl/fl^* mice were significantly larger compared with those in C57BL/6J mice (*P* < 0.05). Each individual data point represents a single unpaired, eye and statistical analysis was carried out using 1-way ANOVA with Tukey’s multiple-comparison test. *P < 0.05, **P < 0.01, ***P < 0.001, and ****P < 0.0001.

To compare our results with previous studies that examined global *Nos3*-KO mice, we measured outflow facility and IOP of *Cre;Nos3^fl/fl^* mice at P90 (21). IOP values of 15.7 ± 1.0, 14.7 ± 1.3, and 18.6 ± 2.7 mmHg (mean ± SD) were recorded from WT mice (*n* = 11 mice), *Cre;Nos3^fl/fl^* mice (*n* = 11), and *Nos3*-KO mice (*n* = 10), respectively (Figure 3A). Consistent with previous studies, outflow facility in *Nos3*-KO mice was significantly reduced compared with that in C57BL/6J mice (*P* = 0.0427, 1-way ANOVA with Tukey’s multiple-comparison test) (20, 21). Outflow facility measurements corresponded with IOP measurements for all strains, with *Cre;Nos3^fl/fl^* mice (*n* = 13 eyes) having the highest geometric mean value at 1.9 (95% CI 1.6, 2.3); WT (*n* = 10 eyes), 1.6 (95% CI 1.4, 1.7); and *Nos3*-KO (*n* = 11 eyes), the lowest, at 1.1 (95% CI 0.8, 1.4) nL/min/mmHg (Figure 3B). Consistent with previous studies, outflow facility in *Nos3*-KO mice was significantly reduced compared with that in C57BL/6J mice (*P* = 0.0427, 1-way ANOVA with Tukey’s multiple-comparison test) (20, 21). Although neither genotype expressed *Nos3* in SC (Figure 2), outflow facility in *Cre;Nos3^fl/fl^* mice was significantly elevated compared with that in *Nos3*-KO mice (*P* = 0.0002). Outflow facility in *Cre;Nos3^fl/fl^* mice was not significantly different from that in C57BL/6J mice (*P* = 0.3646).

Semi-thin methylene blue plastic sections were used to investigate gross morphological differences in cross sections of tissues of the conventional outflow pathway between strains (Figure 3C). No obvious gross morphological alterations were observed in the SC/TM region between strains. Thus, morphology of all 3 mouse genotypes was normal, showing an open iridocorneal angle, a lamellated TM, and a circular SC. To complement histology of iridocorneal angle structures, we imaged each mouse genotype in vivo using spectral domain optical coherence tomography (SD-OCT). The OCT probe was pointed toward the inferior lateral limbus regions of living mice, focusing on the iridocorneal angle, as we previously reported (36). Open iridocorneal angles were observed in all genotypes at P90 (Figure 3D). Since aqueous humor travels in a basal-to-apical direction across SC’s inner wall, unique dome-shaped structures known as giant vacuoles (GVs) often form due to lifting of SC cells from their supporting basement membrane (37). Reduced SC lumen size and GV numbers are associated with increased outflow resistance and elevated IOP in glaucoma (37). Therefore, we quantified both the perimeter of SC (Figure 3E) and number of GVs, normalizing by the inner wall length of SC (Figure 3F) for all strains, using the semi-thin blue cross-sectional images. The mean length of SC lumen was not different between genotypes, measuring 156.7 ± 43.5, 150.0 ± 37.66, and 143.9 ± 28.21 μm (mean ± SD) in WT mice (*n* = 12 eyes, 5–10 sections per eye), *Cre;Nos3^fl/fl^* mice (*n* = 11 eyes, 5–10 sections per eye), and *Nos3*-KO mice (*n* = 8 eyes, 5–10 sections per eye), respectively (Figure 3E). The mean number of GVs was also determined in every section with a clear SC (normalized to inner wall length). There was no difference between genotypes, with measurements of 4.0 ± 2.5, 3.8 ± 2.4, and 2.1 ± 2.0 GVs for WT mice (*n* = 12 eyes, 5–10 sections per eye), *Cre;Nos3^fl/fl^* mice (*n* = 11 eyes, 5–10 sections per eye), and *Nos3*-KO mice (*n* = 8 eyes, 5–10 sections per eye), respectively (Figure 3F), although the GV number trended smaller in *Nos3*-KO mice compared with WT mice (*P* = 0.7468). An example of a GV is shown in Figure 3C (red arrow). The one morphological feature that was different between genotypes was the lumen size of the DVs in *Cre;Nos3^fl/fl^* mice, which appeared enlarged (arrows), compared with both WT and KO mice. The mean circumference of DVs in *Cre;Nos3^fl/fl^* mice (*n* = 12 eyes, 5–10 sections per eye) was significantly larger than in WT mice (*n* = 11 eyes, 5–10 sections per eye) (17.5 ± 8.3 vs. 8.4 ± 4.9 μm, respectively, *P* = 0.0152). *Nos3*-KO mice (*n* = 8 eyes, 5–10 sections per eye) also had enlarged vessels, measuring 13.7 ± 8.6 μm, but the difference compared with WT mice was not significant (Figure 3G). The DVs in *Cre;Nos3^fl/fl^* mice were consistently dilated across all sections; examples are indicated in Figure 3C (black arrows).

### Enlarged DV observed at P90 but not P30 in Cre;Nos3^fl/fl^ mice.

**Figure 4:**
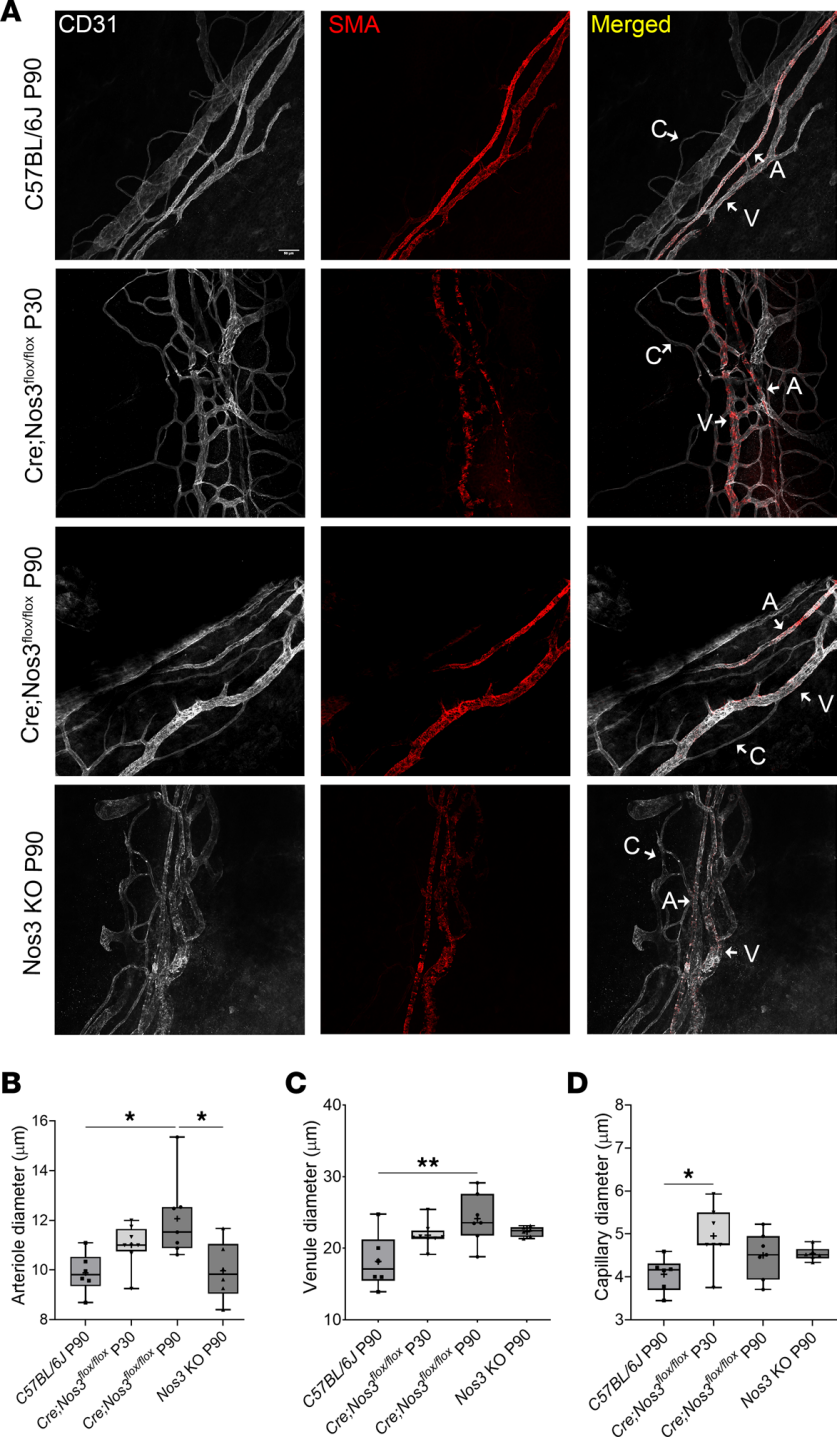
Arterioles and venules enlarge between P30 and P90 in *Cre;Nos3^fl/fl^* mice. (**A**) Representative confocal images of maximum-intensity projection images (taken from *Z*-stacks) shows limbal vasculature from *Cre;Nos3^fl/fl^* mice at P30 and P90, C57BL/6J mice at P90, and *Nos3*-KO mice at P90. Immunofluorescence labeling of CD31 (gray) stains all vasculature (left column) and α-SMA (red) stains all vessels except capillaries (middle column). Merged stains are shown in the right column. Enlargement of both arterioles (A) and venules (V) was visible in outflow tissues of *Cre;Nos3^fl/fl^* mice at P90 compared with C57BL/6J mice. C, capillary. Scale bar: 50 μm. Box-and-whisker plots display summary data (+ representing the mean) showing that (**B**) arteriole diameter was significantly larger in *Cre;Nos3^fl/fl^* mice at P90 (*n* = 7) compared with both C57BL/6J (*n* = 6) and *Nos3*-KO mice, both at P90 (*n* = 6) (*P* = 0.0185 and *P* = 0.0238, respectively; 1-way ANOVA with Tukey’s multiple-comparison test). (**C**) Similarly, venule diameter was significantly larger in *Cre;Nos3^fl/fl^* mice at P90 mice compared with C57BL/6J mice (*P* = 0.0074), but not *Nos3*-KO mice or *Cre;Nos3^fl/fl^* mice at P30 (*n* = 8) (*P* = 0.5407 and *P* = 0.3756, respectively). (**D**) Capillary diameter was significantly larger in *Cre;Nos3^fl/fl^* mice at P30 compared with C57BL/6J animals (*P* = 0.02). Each individual data point represents the average vessel measurement per mouse eye. *P < 0.05 and **P < 0.01; 1-way ANOVA with Tukey’s multiple-comparison test.

Because distal vessel diameter influences outflow function (17, 38–43), we measured limbal vessels that were stained for CD31 (gray) and α-SMA (red) in anterior segment whole mounts by confocal microscopy (Figure 4A). The average of approximately 10 measurements for each arteriole, venule, and capillary was calculated, and the mean vessel diameter for each vessel type is shown for each mouse strain in Figure 4, B–D. In *Cre;Nos3^fl/fl^* mice at P90, CD31 (gray) and α-SMA (red) staining showed arteriole and distal venule enlargement compared with WT mice. In *Cre;Nos3^fl/fl^* mice at P90, mean arteriole diameter (12.1 ± 1.6 μm, *n* = 7) (mean ± SD) was significantly greater than that in C57BL/6J mice (9.9 ± 0.8 μm, *P* = 0.0185, *n* = 6) and *Nos3*-KO mice at P90 (9.9 ± 1.2 μm, *P* = 0.0238, *n* = 6) (Figure 4B). However, in *Cre;Nos3^fl/fl^* mice at P30, the mean arteriole diameter (11.0 ± 0.8 μm, *n* = 8) was not greater than that in WT (*P* = 0.3058) or *Nos3*-KO mice (*P* = 0.3751). In *Cre;Nos3^fl/fl^* mice at P90, the mean distal venule diameter (24.2 ± 3.5 μm, *n* = 7) was also significantly greater than that in C57BL/6J mice (18.2 ± 3.9 μm, *P* = 0.0074, *n* = 6) but not significantly different from that in global *Nos3*-KO mice at P90 (22.3 ± 0.7 μm, *P* = 0.5407, *n* = 6) (Figure 4C). In *Cre;Nos3^fl/fl^* mice at P30, the mean distal venule diameter (21.8 ± 1.8 μm, *n* = 8) was not significantly greater than in WT mice (*P* = 0.0865). Therefore, endothelial postnatal deletion of *Nos3*, occurring around P90, resulted in arteriole and venule enlargement at the same time point in the conventional outflow pathway of *Cre;Nos3^fl/fl^* mice, possibly compensating for loss of *Nos3* in SC. While there is no evidence to suggest that changes to arteriole diameter affect outflow facility, we hypothesize that this expansion in arteriole size, observed in *Cre;Nos3^fl/fl^* mice at P90, was due to NO dysregulation in arteriole endothelia. We also measured collector channel diameter, and there was no significant difference in diameter between strains (Supplemental Figure 4).

There were 2 additional interesting findings from this set of experiments. The first was that the α-SMA signal appeared to be weaker in both *Cre;Nos3^fl/fl^* mice at P30 and *Nos3*-KO mice compared with C57BL/6J and *Cre;Nos3^fl/fl^* mice at P90 (Figure 4A). Second, mean capillary diameter (5.0 ± 0.7 μm, *n* = 7) was significantly greater in *Cre;Nos3^fl/fl^* mice at P30 than in C57BL/6J mice at P90 (4.1 ± 0.4 μm, *P* = 0.02, *n* = 6). However, there was no difference in capillary diameter between *Cre;Nos3^fl/fl^* mice at P90 and C57BL/6J control mice (*Cre;Nos3^fl/fl^* mice P90 4.5 ± 0.5 μm, WT 4.1 ± 0.4 μm, and *Nos3*-KO 4.5 ± 0.2 μm, *n* = 6–7) (Figure 4D).

### ELAM1 expression, recruitment of macrophages, and upregulation of NOS2 expression in the DV of Cre;Nos3^fl/fl^ mice between P60 and P90.

**Figure 5:**
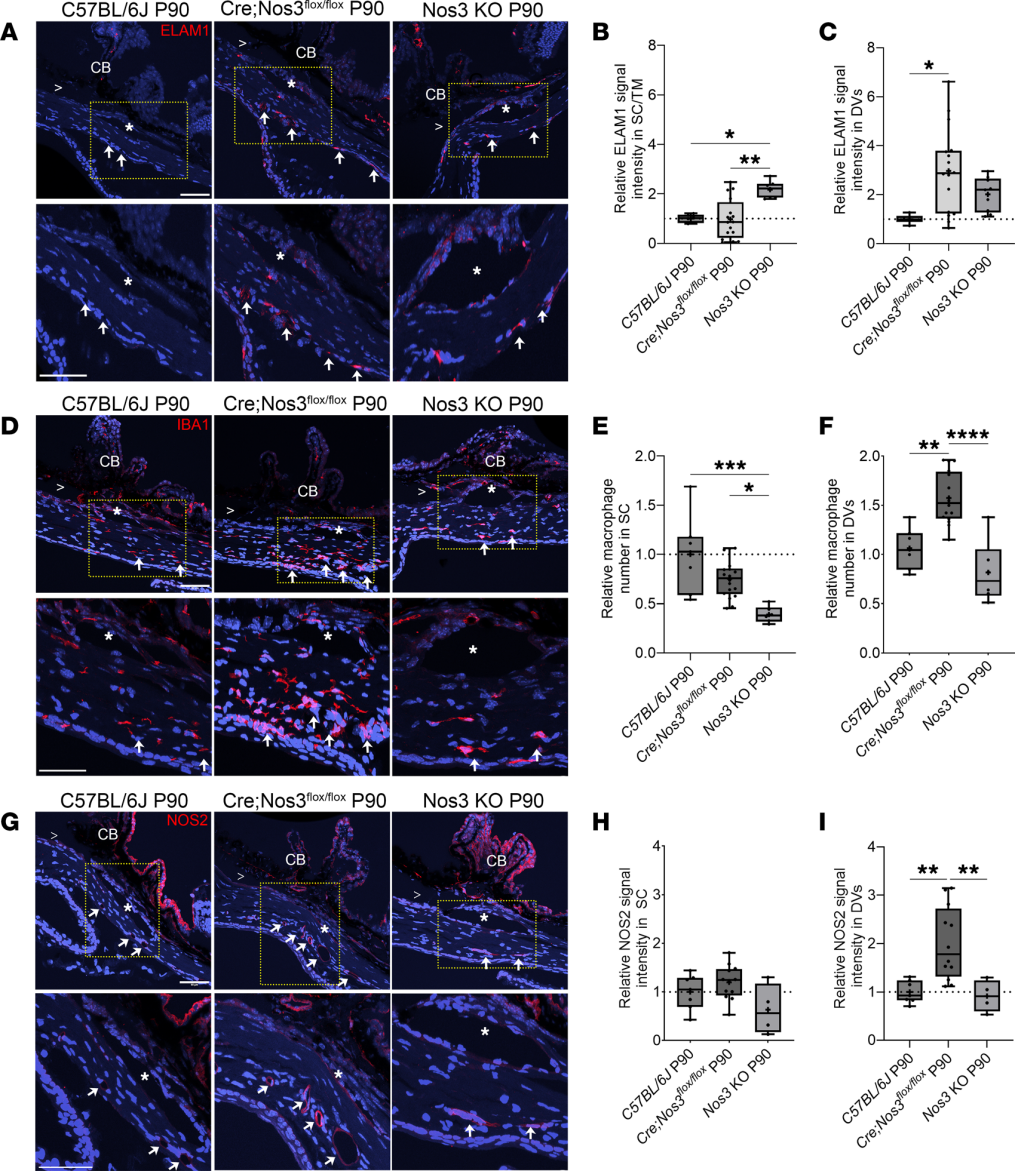
Increased ELAM1 protein expression coincides with recruitment of IBA1^+^ macrophages to and NOS2 expression in the DV region of *Cre;Nos3^fl/fl^* mice at P90. (**A**) Representative images showing ELAM1 expression (red) in C57BL/6J, *Cre;Nos3^fl/fl^*, and KO mice at P90. R, retina; >: open iridocorneal angle. Arrows: DV; asterisks: SC. Images are representative of *n* = 3–6 eyes; 5–10 sections per eye for each time point. Scale bar: 50 μm. (**B**) There was a significant increase in ELAM1 expression in the SC/TM region in *Nos3*-KO mice compared with both C57BL/6J (*P* = 0.0164) and *Cre;Nos3^fl/fl^* mice (*P* = 0.0014). (**C**) In contrast, there was a significant increase in ELAM1 in the DV region (arrows) in *Cre;Nos3^fl/fl^* mice compared with C57BL/6J mice (*P* = 0.0398). (**D**) Representative images showing IBA1^+^ macrophages (red) in the SC region (*) and surrounding DV (arrows) of C57BL/6J, *Cre;Nos3^fl/fl^*, and KO mice at P90. An increased number of IBA1^+^ macrophages was observed around the DV (arrows) of *Cre;Nos3^fl/fl^* mice, while (**E**) a decreased number of IBA1^+^ macrophages was observed around the SC region of *Cre;Nos3^fl/fl^* mice and KO mice compared with C57BL/6J mice. (**F**) A significant increase in the number of IBA1^+^ macrophages was observed around the DV of *Cre;Nos3^fl/fl^* mice compared with both C57BL/6J and KO mice. (**G**) Representative images showing NOS2 protein expression (red) in the SC region (*) and surrounding DV (arrows) of C57BL/6J, *Cre;Nos3^fl/fl^*, and KO mice at P90. (**H**) There was no significant difference in relative NOS2 signal intensity in the SC region between *Cre;Nos3^fl/fl^*, C57BL/6J, and KO mice at P90. (**I**) Instead, there was a significant increase in NOS2 signal intensity observed in the DV of *Cre;Nos3^fl/fl^* mice compared with both C57BL/6J and KO mice. Each individual data point represents a single unpaired eye. *P < 0.05, **P < 0.01, ***P < 0.001, and ****P < 0.0001; 1-way ANOVA with Tukey’s multiple-comparison test.

To examine the mechanism of macrophage recruitment to DVs, we probed for endothelial cell–leukocyte adhesion molecule (ELAM1). ELAM1 mediates an endothelial cell–dependent mechanism for the regulation of leukocyte–vessel wall adhesion (44, 45). Consistent with its role as a biomarker for primary open angle glaucoma (POAG) (46), we observed a significant increase in ELAM1 protein expression in the SC/TM region of *Nos3*-KO mice at P90 (2.2 ± 0.3, *n* = 7, Figure 5, A and B) compared with both C57BL/6J mice at P90 (1.0 ± 0.2, *P* = 0.0167, 1-way ANOVA with Tukey’s multiple-comparison test, *n* = 6) and *Cre;Nos3^fl/fl^* mice at P90 (1.0 ± 0.8, *P* = 0.0014, *n* = 16). In contrast, we observed no difference in ELAM1 expression in the DV region of *Nos3*-KO mice (2.0 ± 0.7, *n* = 9, Figure 5, A and C) compared with either C57BL/6J (1.0 ± 0.2, *P* = 0.6559, *n* = 6) or *Cre;Nos3^fl/fl^* mice (3.0 ± 1.7, *P* = 0.4932, *n* = 16). We did, however, observe a significant increase in ELAM1 expression in the DV region between C57BL/6J and *Cre;Nos3^fl/fl^* mice (*P* = 0.0398, Figure 5, A and C) at P90, the time when we observed significantly more macrophages in the DVs.

NOS2 is inducible and can be expressed by both endothelia and macrophages. We stained outflow tissues of C57BL/6J, *Cre;Nos3^fl/fl^*, and *Nos3*-KO mice with antibodies that recognize a general macrophage marker, ionized calcium binding adaptor molecule 1 (IBA1). Representative images of C57BL/6J, *Cre;Nos3^fl/fl^* mice, and KO mice at P90 are shown in Figure 5D; P10–P60 *Cre;Nos3^fl/fl^* mice are shown in Supplemental Figure 6. IBA1^+^ macrophages (red) were costained with α-SMA (green) in order to identify the TM and CD31 (magenta) to identify endothelia of both SC (asterisk) and surrounding DV (arrows) (Supplemental Figure 6). Relative macrophage abundance near the SC region and surrounding DV was calculated using the circumferential size of SC lumen and surrounding DV for each sample and counting the IBA1^+^ macrophages (Figure 5, E and F). A significant increase in the number of IBA1^+^ macrophages was observed near the DV region in *Cre;Nos3^fl/fl^* mice only at P90 compared with both C57BL/6J mice at P90 (1.6 ± 0.3, *n* = 16 vs. 1.0 ± 0.2, respectively; *n* = 7, *P* = 0.0029, 1-way ANOVA with Tukey’s multiple-comparison test) and *Nos3*-KO mice at P90 (1.6 ± 0.3, *n* = 16 vs. 0.8 ± 0.3, *n* = 6, *P* < 0.0001) (Figure 5F). However, the number of macrophages near the SC region was not different between WT and *Cre;Nos3^fl/fl^* mice (Figure 5E). Interestingly, there was a significant decrease in relative number of macrophages near the SC region in *Nos3*-KO mice at P90 compared with both C57BL/6J mice at P90 (0.4 ± 0.08, *n* = 6 vs. 1.0 ± 0.4, *n* = 7, *P* = 0.0002) and *Cre;Nos3^fl/fl^* mice at P90 (0.4 ± 0.08, *n* = 6 vs. 0.8 ± 0.2, *n* = 18, *P* = 0.0169) (Figure 5E).

NOS isoforms have the potential to compensate for each other when one is knocked out. To check for potential compensatory changes in *Nos1* and *Nos2* due to postnatal deletion of *Nos3*, we carried out RNAscope and IHC on tissue sections of the outflow pathway from *Cre;Nos3^fl/fl^*, C57BL/6J, and *Nos3*-KO mice. There was no difference in NOS1 expression at either the SC/TM region or distal vessel region in any of the strains, across all ages (Supplemental Figure 7). Similar results were observed for *Nos1* mRNA using RNAscope (Supplemental Figure 5). Results showed no difference in relative NOS2 signal intensity in the SC region between C57BL/6J mice at P90 (1.0 ± 0.4, *n* = 7), *Cre;Nos3^fl/fl^* mice at P90 (1.2 ± 0.4, *n* = 12), and KO mice at P90 (0.6 ± 0.5, *n* = 4, Figure 5, G and H). However, there was a significant increase in relative NOS2 signal intensity in the distal vascular region of *Cre;Nos3^fl/fl^* mice at P90 (2.0 ± 0.8, *n* = 12) compared with both C57BL/6J mice at P90 (1.0 ± 0.2, *n* = 7, *P* = 0.0024, 1-way ANOVA with Tukey’s multiple-comparison test) and KO mice at P90 (0.9 ± 0.3, *n* = 4, *P* = 0.0079, Figure 5I). NOS2 expression in the distal vessels of *Cre;Nos3^fl/fl^* mice at any other time point (except P90), was not different from that of WT mice (Supplemental Figure 8). Similar results were observed for *Nos2* mRNA using RNAscope (Supplemental Figure 5).

**Figure 6:**
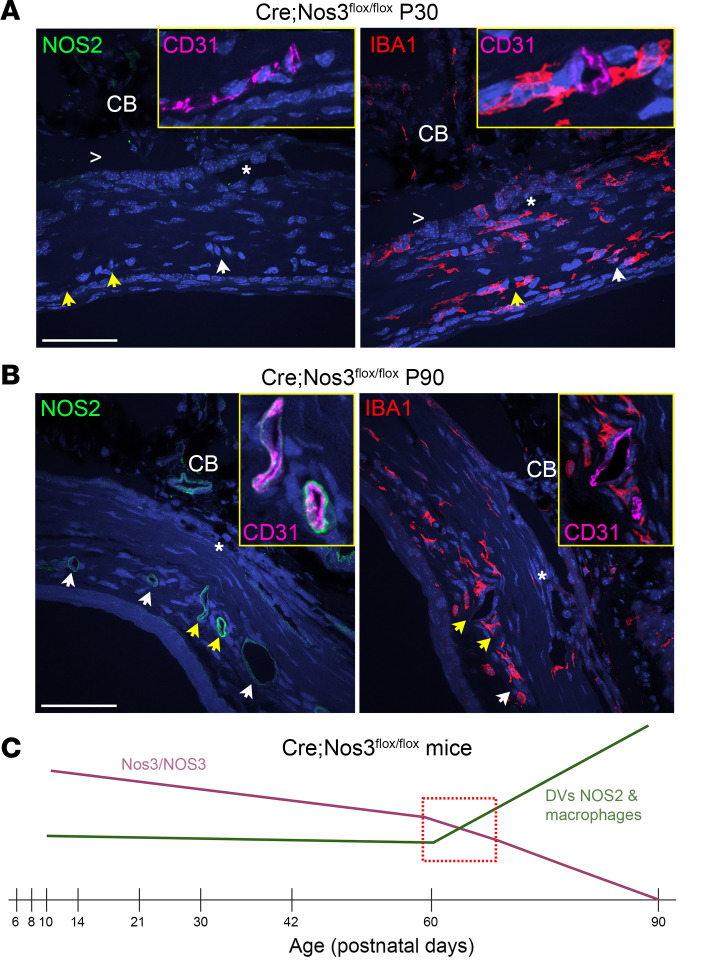
Induction of NOS2 expression in distal endothelia of *Cre;Nos3^fl/fl^* mice occurs between P30 and P90. (**A**) In *Cre;Nos3^fl/fl^* mice at P30, NOS2 expression (green) was not observed in either the SC region (*) or DV region (arrows). DVs appeared to be normal in size, and IBA1^+^ macrophages (red) were distributed evenly throughout the outflow pathway. Yellow arrows point to DVs, which are shown costained with CD31 (magenta) in the inset. (**B**) At P90, enlarged DVs were observed and were coincident with NOS2 staining (green), resembling the staining pattern of the endothelial marker CD31 (magenta). Image shown is enlarged version of the corresponding image in Figure 5G. IBA1^+^ macrophages (red) were more abundant in the DV region (yellow box). >: open iridocorneal angle; asterisks: SC; arrows: DV. Scale bars: 50 μm. Both NOS2 and IBA1 antibodies were raised in rabbit, so sequential sections were used for IHC. (**C**) Schematic summary showing that *Nos3* was gradually deleted over time in these mice (pink) from P10 to P90. At P90, *Nos3* was completely deleted, so that these mice resembled global KO mice. Between P60 and P90, NOS2 expression was obvious in the endothelia of the DVs, and macrophages were more abundant in the region and surrounded the endothelial DVs (green). We hypothesize that when a certain threshold of *Nos3* deletion has been reached (red box), macrophages infiltrate and NOS2 is induced in the DVs, which enlarge to compensate for loss of NOS2 in SC and DVs.

In order to more clearly visualize NOS2 expression in *Cre;Nos3^fl/fl^* mice at P90, we examined NOS2 expression in relation to macrophage (IBA1) versus endothelial (CD31) markers in the outflow regions (Figure 6). As before, confocal imaging showed no detectible NOS2 expression in the SC or DV region at P30 in *Cre;Nos3^fl/fl^* mice (Figure 6A). In contrast, NOS2 expression was apparent in the enlarged DVs (Figure 6B), displaying a circular, endothelial pattern. Importantly, NOS2 expression colocalized with CD31, but not with IBA1. Interestingly, IBA1^+^ macrophages were uniformly present in *Cre;Nos3^fl/fl^* mice at P30 throughout the outflow pathway, similar to those in C57BL/6J mice (Figure 5D). At P90 however, macrophages appeared to be more abundant in the DV region compared with the SC/TM region in *Cre;Nos3^fl/fl^* mice. In summary, *Nos3* appears to gradually decrease overtime in *Cre;Nos3^fl/fl^* mice from P10 to P90, with complete loss of *Nos3* observed at P90. Between P60 and P90, enlargement of DVs was observed to coincide with NOS2 expression apparent in the DVs themselves, as well as recruitment of macrophages to the DV region (Figure 6C).

### Inhibition of iNOS elevated IOP and reduced vessel diameter in Cre;Nos3^fl/fl^ mice.

**Figure 7:**
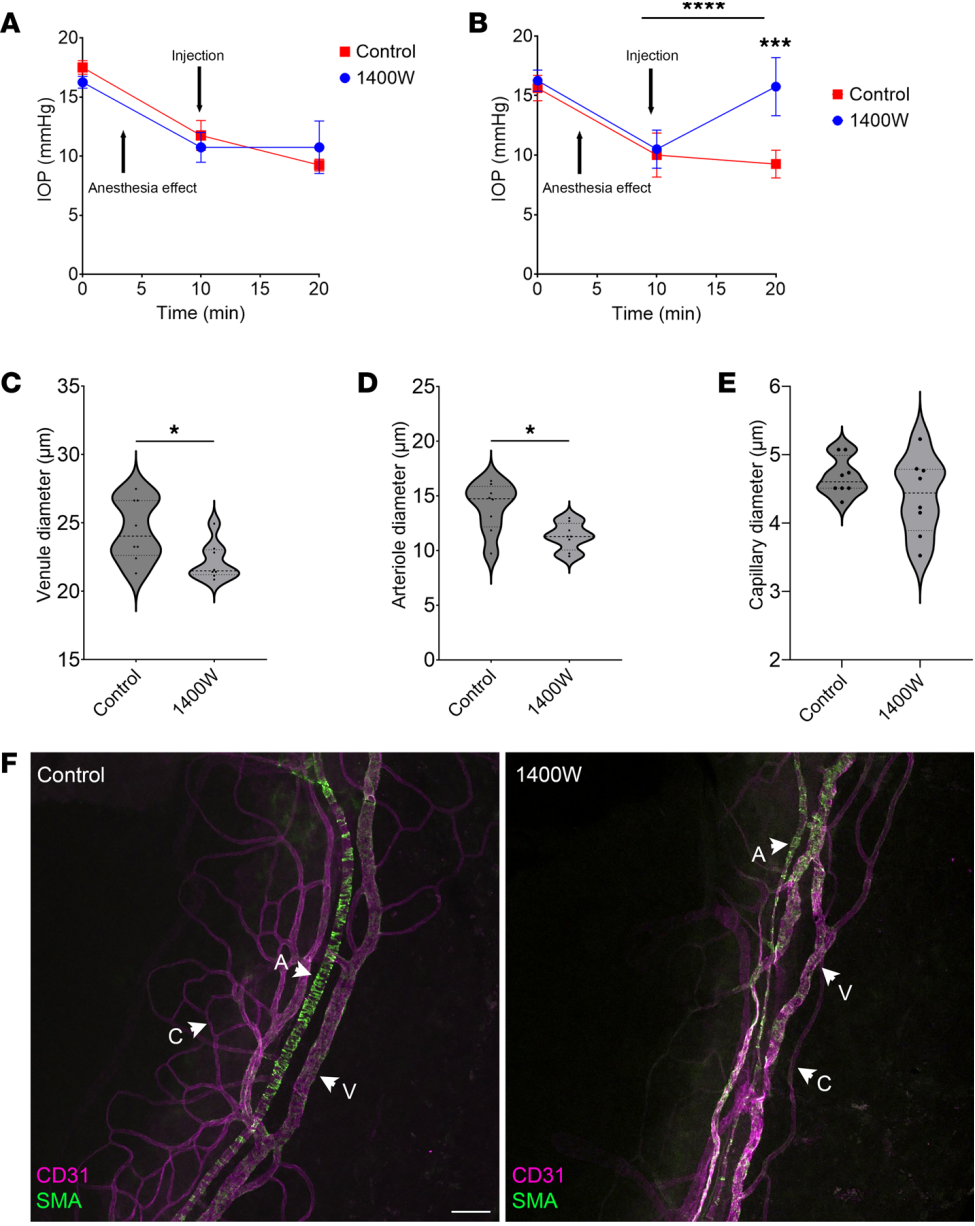
Inhibition of NOS2 resulted in elevated IOP and decreased distal vessel diameter in *Cre;Nos3^fl/fl^* but not in C57BL/6J mice. (**A**) In C57BL/6J mice, IOP measurements were taken before injection and 10 minutes after anesthesia, after a ketamine/xylazine-induced IOP decrease. Ten minutes following 1400W injection, IOP was taken a third time. There was no significant difference between control (red) and 1400W-treated eyes (blue; *P* = 0.2665). (**B**) In *Cre;Nos3^fl/fl^* mice, IOP measurements were taken before injection and 10 minutes after anesthesia, after a ketamine/xylazine-induced IOP decrease. Ten minutes after injection of PBS or 1400W, IOP was measured a third time. Results show no effect of PBS injection on IOP in control eyes (red). 1400W-treated eyes (blue), however, had a significant increase in IOP 10 minutes after injection (*P* < 0.0001). Distal vessel characterization was then carried out and vessel diameter measured for all *n* = 8 pairs of eyes. (**C**) A significant decrease in venule diameter was observed in 1400W-treated compared with control eyes (*P* = 0.0229). (**D**) A significant difference in arteriole diameter was also observed between control and 1400W-treated eyes (*P* = 0.0108). (**E**) There was no difference in capillary diameter (*P* = 0.2136). Each data point represents the average measurement per eye from all 4 quadrants of *n* = 8 eyes. (**F**) Representative images showing venules (V), arterioles (A), and capillaries (C) of the DV in control and 1400W-treated eyes of *Cre;Nos3^fl/fl^* mice at P90. The DV was stained with both the endothelial marker CD31 (magenta) and the α-SMA marker (green). Each individual data point represents a single unpaired eye.*P < 0.05, **P < 0.01, and ****P < 0.0001, 1-way ANOVA with Tukey’s multiple-comparison test. Scale bar: 50 μm.

C57BL/6J mice at P90 were anesthetized with ketamine/xylazine. IOP measurements were taken as soon as the mice were under anesthesia, and mean IOP values of 17.5 ± 0.6 mmHg and 16.3 ± 0.5 mmHg were recorded in control and treated eyes, respectively (Figure 7A). IOP measurements were taken again once IOP had significantly decreased due to the effects of ketamine/xylazine (10 minutes), showing mean IOP values of 11.8 ± mmHg and 10.8 ± 1.3 mmHg for control and treated eyes, respectively (*P* = 0.0022, 1-way ANOVA, *n* = 4 pairs of eyes; Figure 7A). These mice received a 10 μL subconjunctival injection of 1400W iNOS inhibitor (50 μM) in one eye and a sham injection of PBS in the contralateral control eye. Ten minutes after injection, IOP measurements were taken a third time. Mean IOP values of 9.3 ± 0.5 mmHg and 10.8 ± 2.2 mmHg were observed in control and 1400W-treated eyes, respectively (*P* = 0.2665, paired *t* test, *n* = 4, Figure 7A).

The same experiment was carried out on *Cre;Nos3^fl/fl^* mice at P90. Initial IOP measurements of 15.6 ± 1.1 mmHg and 16.2 ± 0.9 mmHg (*n* = 8) were measured for both control and treated eyes, respectively (Figure 7B). IOP measurements were taken again once IOP had significantly decreased, showing mean IOP values of 10.0 ± 1.9 mmHg and 10.5 ± 1.6 mmHg for both control and treated eyes, respectively (*P* < 0.0001, 1-way ANOVA with Tukey’s multiple-comparison test, *n* = 8 pairs of eyes, Figure 7B). Following injection of 1400W iNOS inhibitor (50 μM) in treated eyes and a sham injection of PBS in the contralateral control eye, IOP was measured for a third time. Mean IOP values of 9.3 ± 1.2 mmHg and 15.8 ± 0.9 mmHg were observed in control and 1400W treated eyes, respectively (*P* = 0.0001, paired *t* test, *n* = 8). Coincident with the 1400W-mediated increase in IOP, both venules and arterioles were smaller in size in 1400W treated eyes compared with control eyes. 1400W treated eyes had a mean venule diameter of 24.5 ± 2.3 μm compared with 22.1 ± 1.4 μm in control eyes (*P* = 0.0229, paired *t* test, *n* = 8 pairs of eyes, Figure 7C). Mean arteriole diameter of 11.3 ± 1.2 μm was observed in 1400W treated eyes compared with 14.0 ± 2.3 μm in control eyes (*P* = 0.0108, *n* = 8 pairs, Figure 7D). There was no significant difference in capillary diameter between groups with mean capillary diameter of 4.4 ± 0.6 μm in 1400W treated eyes and 4.7 ± 0.3 μm in control eyes (*P* = 0.2136, *n* = 8 pairs, Figure 7E). Representative images of control and 1400W-treated *Cre;Nos3^fl/fl^* eyes are shown below (Figure 7F).

## Discussion

An unexpected, but fortuitous, finding was that the endothelial enhancer of the stem cell leukemia (SCL) locus in the *Cre;Nos3^fl/fl^* mice began to drive Cre-ER^T^ recombinase expression in the absence of tamoxifen treatment at P10 (Figure 1). Cre-ER^T^ expression gradually increased with the age of the mice until P90, at which time *Nos3* mRNA and NOS3 protein levels in the SC and DV regions of *Cre;Nos3^fl/fl^* mice were indistinguishable from those of global *Nos3*-KO mice (Figure 2). DV enlargement was observed in *Cre;Nos3^fl/fl^* mice at P90, but not P30 (Figure 4), which coincided with increased expression of ELAM1 and NOS2 in the distal endothelia and recruitment of IBA1^+^ macrophages to this region (Figure 6). Increased numbers of monocytes/macrophages in outflow tissues, following selective laser trabeculoplasty, have previously been shown to correlate with increased outflow facility and SC conductivity, highlighting the important role immune cells play in outflow homeostasis (47). In the Cre;Nos3^fl/fl^ mice used in our study, these postnatal adjustments in conventional outflow physiology resulted in homeostatic maintenance of outflow facility and IOP, unlike in the ocular hypertensive global Nos3-KO mice (Figure 3). Reproductive and developmental issues that were observed in *Nos3*-KO mice (Supplemental Table 1) were also not present in *Cre;Nos3^fl/fl^* mice and further motivated the use of *Cre;Nos3^fl/fl^* mice.

### The Cre-lox system.

The Cre-lox system has become a powerful tool in mouse genetic studies that allows for selected targeted gene expression (48), resulting in the generation of conditional KO or knock-in mice, and for remarkable control over gene expression (49–51). However, the Cre-lox system is not without its flaws. Many researchers have reported issues when using the Cre-lox system, including spontaneous recombination of Cre activity, Cre activity in nontargeted genes, and Cre toxicity (48, 52–57). As in this study, other researchers have reported spontaneous Cre-ER^T^ activity in the absence of tamoxifen treatment (54, 58, 59). Many Cre-ER^T^ lines have been reported to have spontaneous Cre recombination regardless of its specific promoter (58, 60–62). Spontaneous Cre activity can be a confounding factor without the use of appropriate controls. This spontaneous Cre recombination was verified in the present study using 2 independent reporter mouse strains, *R26R* and *Ai9*. We quantified *Nos3*/NOS3 expression at various ages (P10–P90) using 3 separate methods (RNAscope, IHC, and qPCR) and included appropriate controls (C57BL/6J and global KO mice). We also investigated the possibility of Cre-ER^T^ toxicity and tested the *end-SCL-Cre-ER^T^* mice in control experiments. Both IOP and facility measurements were similar to those of C57BL/6J mice, with no gross morphological differences observed in conventional outflow tissue, including surrounding DV (Supplemental Figure 9). Thus, we are confident that the results observed in the *Cre;Nos3^fl/fl^* mice were not due to the effects of Cre-ER^T^ recombinase itself but due to the gradual suppression of *Nos3* over time.

### The gradual deletion of Nos3 over time in Cre;Nos3^fl/fl^ mice.

Three separate methods were used to monitor the timing of *Nos3* expression and deletion. Quantitative PCR, RNAscope, and IHC showed “global *Nos3*-KO” levels of *Nos3* mRNA and NOS3 protein in *Cre;Nos3^fl/fl^* mice by P90. Importantly, remarkable decreases in *Nos3* expression did not occur in mice until after SC was fully developed, around P17–P21 (35). Last, we observed when genotyping *Cre;Nos3^fl/fl^* mice that *Nos3* gene expression (alongside Cre-ER^T^ and *flox/flox*) were present in all mice used in this study (Supplemental Figure 12). Single-cell transcriptomics of outflow tissues from developing C57BL/6J mice demonstrated *Nos3* expression in vascular progenitor cells and blood endothelial cells (BECs) as early as day 2 and SC (immature state) at day 6.5 (Supplemental Figure 13) – suggesting a role for *Nos3* in SC during development. Together, these results and the LacZ staining for Cre-ER^T^ expression support the concept that spontaneous Cre-ER^T^ recombinase activity was a postnatal event, unlike the germline deletion observed in the *Nos3*-KO mice. Importantly, our results were consistent with other studies showing decreased outflow facility, elevated IOP, and normal gross morphology of the SC and TM in global *Nos3*-KO mice, which was different from the *Cre;Nos3^fl/fl^* and C57BL/6J WT mice (20, 21).

### The upregulation of ELAM1 in the DV of Cre;Nos3^fl/fl^ mice.

ELAM1 expression has been identified as a disease marker for glaucoma (46). The presence of ELAM1 in the SC/TM region of global *Nos3*-KO mice is consistent with evidence in humans showing that polymorphisms in *Nos3* may be associated with the disease (24, 63–65). As is the case in glaucoma, global *Nos3*-KO mice have elevated IOP and reduced outflow facility. The *Cre;Nos3^fl/fl^* mice, however, did not have elevated IOP and/or reduced outflow facility. Unlike in global *Nos3*-KO mice, ELAM1 expression was not observed in the SC/TM but instead in the DV region of *Cre;Nos3^fl/fl^* mice (Figure 5). Since ELAM1 expression has been associated with the recruitment of leukocytes to blood vessel walls (44), we hypothesized that the increased numbers of IBA1^+^ macrophages in the DV region, compared with the SC/TM region, of *Cre;Nos3^fl/fl^* mice at P90 were due to upregulation of ELAM1 (Figure 5). It has been suggested that ELAM1 activation is due to cytokine release and not changes resulting from shear stress (46) and may explain why ELAM1 is not observed in the SC/TM region in *Cre;Nos3^fl/fl^* mice.

### Compensatory effects of NOS isoforms.

NOS isoforms compensate for one another in single, germline KO mice. For example, *Nos2* expression in spinal cords was significantly increased in both *Nos1*- and *Nos3*-deficient mice (66). Another study reported compensation by *Nos2* to preserve endothelium-dependent relaxation in the coronary circulation in both heterozygous and homozygous *Nos3*-KO mice (67). Potentially relevant to the role of NOS in SC function, *Nos1* was observed to be upregulated and responsive to shear stress in coronary artery endothelial cells (68). To see whether there were similar compensatory changes in *Cre;Nos3^fl/fl^* mice (P10–P90), we screened the SC and DV region for changes in *Nos1*/NOS1 and *Nos2*/NOS2, measuring the mRNA expression (using RNAscope; Supplemental Figure 5) and protein expression (Supplemental Figures 7 and 8) of both. While we observed no difference in *Nos1* mRNA or NOS1 protein expression in either the SC or DV region in *Cre;Nos3^fl/fl^* mice compared with both C57BL/6J and KO mice at P90 (Supplemental Figure 7), there was a significant increase in both *Nos2* mRNA and NOS2 protein expression near the DV region between P60 and P90 (Figure 5C and Supplemental Figure 8). Confocal imaging showed no detectible NOS2 expression in SC or DV region at P30 in *Cre;Nos3^fl/fl^* mice (Figure 6A). In contrast, NOS2 expression was apparent in the enlarged DVs (Figure 6B) displaying a circular, endothelial pattern. Importantly, NOS2 expression colocalized with CD31, but not with IBA1.

### Macrophages in the outflow pathway.

Interestingly, IBA1^+^ macrophages were uniformly present in *Cre;Nos3^fl/fl^* mice at P30 throughout the outflow pathway, similar to those in C57BL/6J mice (Figure 5D). At P90, however, macrophages appeared to be more abundant in the DV region compared with the SC/TM region in *Cre;Nos3^fl/fl^* mice. In summary, *Nos3* appears to gradually decrease over time in *Cre;Nos3^fl/fl^* mice from P10 to P90, with complete loss of *Nos3* observed at P90. Between P60 and P90, enlargement of DVs was observed to coincide with NOS2 expression apparent in the DVs themselves, as well as recruitment of macrophages to the DV region (Figure 6C). Single-cell transcriptomics showed that *Nos2* was not highly expressed in BECs, SC endothelial cells (SECs), or lymphatic endothelial cells (LECs) (Supplemental Figure 13) in outflow tissues of C57BL/6J mice. Therefore, this increase in *Nos2*/NOS2 observed in the DVs of *Cre;Nos3^fl/fl^* mice (Figure 6) is even more compelling. Since these compensatory effects were not observed in global KO mice, we hypothesize that the gradual decrease in *Nos3* expression in *Cre;Nos3^fl/fl^* mice triggered homeostatic adjustments in outflow that included recruitment of macrophages and induction of NOS2 by DVs. Portions of DVs contain α-SMA (69–72), meaning they are likely vasoregulated and responsive to NO, explaining vasodilation in *Cre;Nos3^fl/fl^* mice. To our knowledge, this is the first observation of the involvement of macrophages in compensatory changes due to NOS deletion.

### Targeted downregulation of iNOS in the distal endothelia of Cre;Nos3^fl/fl^ mice.

To mechanistically test our hypothesis that IOP maintenance was achieved in the face of postnatal deletion of *Nos3* due to upregulation of NOS2 in the distal endothelia, we directly inhibited iNOS — using the iNOS inhibitor 1400W — in the DVs of *Cre;Nos3^fl/fl^* mice. Consistent with this hypothesis, iNOS inhibition in the DV resulted in decreased vessel diameter and elevated IOP in *Cre;Nos3^fl/fl^* mice at P90 (Figure 7). This result suggested that the compensatory effects of NOS2 expression by DVs was eliminated with iNOS inhibition. As a control, we treated C57BL/6J mice with 1400W subconjunctively but saw no effect on IOP (Figure 7A). This result aligned with our NOS2 staining of C57BL/6J mice, where we observed no NOS2 in the cells of the conventional outflow tract. As a second control, we treated C57BL/6J mice having normal NOS3 expression in their DVs with subconjunctival l-NAME. Results showed a significant decrease in DV size and subsequent increase in IOP (Supplemental Figure 10). To increase confidence in our method used to characterize distal vessels, we carried out an experiment using a rho kinase inhibitor (Rhopressa) to lower IOP and increase distal venule diameter following immersion fixation (Supplemental Figure 11).

### Clinical relevance of NO.

There is an abundance of evidence that demonstrates the clinical relevance of NO signaling on outflow function and thus IOP control. First, polymorphisms in *Nos3* or *Cav1* (a gene that encodes a scaffolding protein that sequesters eNOS) impart risk for ocular hypertension and glaucoma in multiple studies involving different populations (26, 73–85). In addition, indicators of NO signaling were significantly lower in aqueous humor from glaucoma patients in 2 different studies (86, 87). In terms of protection from glaucoma, higher dietary nitrate and green leafy vegetable intake associated with a lower glaucoma risk in a large study involving approximately 100,000 people (88). Due to evidence of defective NO signaling in glaucoma and lower glaucoma risk with nitrate intake, NO donors have become attractive molecules for development and use as IOP-lowering medications. Latanoprostene bunod, which was approved by the US FDA to treat ocular hypertension (89, 90), is a combination drug that includes a first-line glaucoma medication (latanoprost, a prostaglandin F2α analog) and an NO-donating group. A second drug, NCX 470, is also a combination drug that has a different prostaglandin as its core, bimatoprost with a NO-donating group (2). Phase III clinical trials show more effective IOP-lowering capabilities across all time points using NCX 470 0.1% compared with latanoprost (91). Furthermore, nitrovasodilators are effective at lowering IOP by decreasing outflow resistance in both rabbits (92) and monkeys (93). Results from our study emphasize the importance of NO supplementation, here by distal endothelial cells, to restore conventional outflow function, which could motivate novel ways to think about treating ocular hypertension and glaucoma.

## Methods

Detailed methods can be found in Supplemental Methods.

### Sex as a biological variable.

We checked both males and females for spontaneous Cre-ER^T^ recombination, thinking estrogen in female mice could contribute to Cre-ER^T^ activation. However, we did not see a difference between males and females. Therefore, sex was not considered as a biological variable in this study.

### Animal studies.

Mice with endothelial cell–specific, tamoxifen-inducible Cre-ER^T^ targeting *Nos3* (*Endo-SclCre-ER^T^;Nos3^fl/fl^* mice) (94) were sent from Vanderbilt University to Duke University. *Cre;Nos3^fl/fl^* mice were bred with 2 independent reporter strain mice, *R26R* (no. 003474) and *Ai9* tdTomato (no. 007909), purchased from The Jackson Laboratory, to characterize Cre-ER^T^ expression. C57BL/6J mice, *Nos3*-KO mice (no. 002684), and *end-SCL-Cre-ER^T^* mice (no. 037467) were all used as control animals. More detailed information is presented in Supplemental Methods.

### Primers.

Primers are listed in Table 1.

### Statistics.

Individual sample sizes are specified in each figure legend. Statistical analysis was carried out using GraphPad prism v.10 (GraphPad Software). Comparisons between groups were assessed by paired or unpaired *t* tests and 1-way ANOVA with Tukey’s multiple-comparison test. A *P* value less than 0.05 was considered statistically significant, and data are presented as mean ± SD, unless otherwise stated.

### Study approval.

All studies involving animals were compliant with the Association for Research in Vision and Ophthalmology (ARVO) Statement for the Use of Animals in Ophthalmic and Vision Research and the NIH *Guide for the Care and Use of Laboratory Animals* (National Academies Press, 2011). All mouse experiments were performed according to procedures approved by the Duke University Animal Care and Use Committee.

### Data availability.

Values for all data points in graphs are reported in the Supporting Data Values file.

## Author contributions

RAK led the studies and was directly involved in all experiments. WDS oversaw the studies. RAK, MSK, MHE, GL, and KMP conducted experiments and acquired data. TT provided the mice used in the study. RB and SWMJ provided single-cell expression data. RAK, MSK, MHE, KMP, GL, ERT, RB, SJ, DRO, and WDS interpreted data. RAK and WDS wrote the manuscript. All authors discussed the results and commented on the manuscript.

## Supplementary Material

Supplemental data

Supporting data values

## Figures and Tables

**Table 1:**
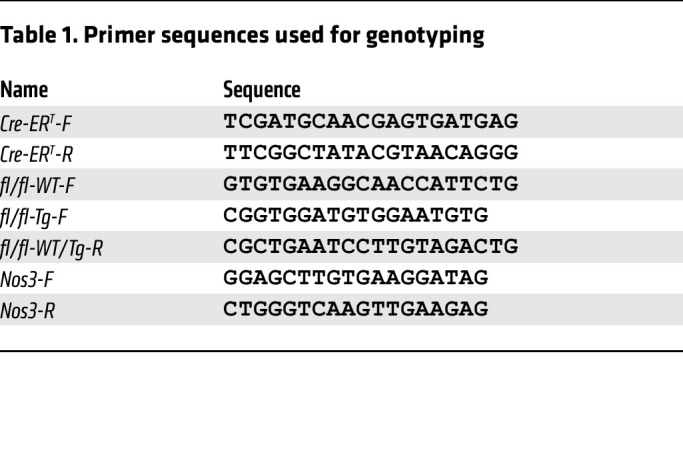
Primer sequences used for genotyping

## Version 1 02/11/2025

In-Press Preview

## Version 2 04/01/2025

Electronic publication
