# Supplementary material for: Endothelial cell–specific postnatal deletion of Nos3 preserves intraocular pressure homeostasis via macrophage recruitment and NOS2 upregulation
Source: J Clin Invest. 2025 Feb 11;135(7):e183440. doi: 10.1172/JCI183440 (PMC11957705; doi:10.1172/JCI183440)
Supplement: Supplemental data [file jci-135-183440-s115.pdf]

Endothelial-specific postnatal deletion of Nos3 preserves intraocular pressure homeostasis via macrophage recruitment and NOS2 upregulation

Ruth A. Kelly[1], Megan S. Kuhn[1], Ester Reina-Torres[2], Revathi Balasubramanian[3], Kristin M. Perkumas[1], Guorong Li[1], Takamune Takahashi[4], Simon W.M. John[3], Michael H. Elliott[5, 6*], Darryl R. Overby[4*], W. Daniel Stamer[1*]

[1]Department of Ophthalmology, Duke Eye Center, Duke University, Durham, NC, USA.

[2]Deparment of Bioengineering, Imperial College London, London, UK.

[3]Department of Ophthalmology, Columbia University, New York, NY, USA.

[4]Division of Nephrology and Hypertension, Vanderbilt University, Nashville, TN, USA.

[5]Department of Ophthalmology, Dean McGee Eye Institute University of Oklahoma Health Sciences Center, Oklahoma City, OK, USA.

[6]Department of Physiology, University of Oklahoma Health Sciences Center, Oklahoma City, OK, USA.

*MHE DRO and WDS are co-corresponding authors.

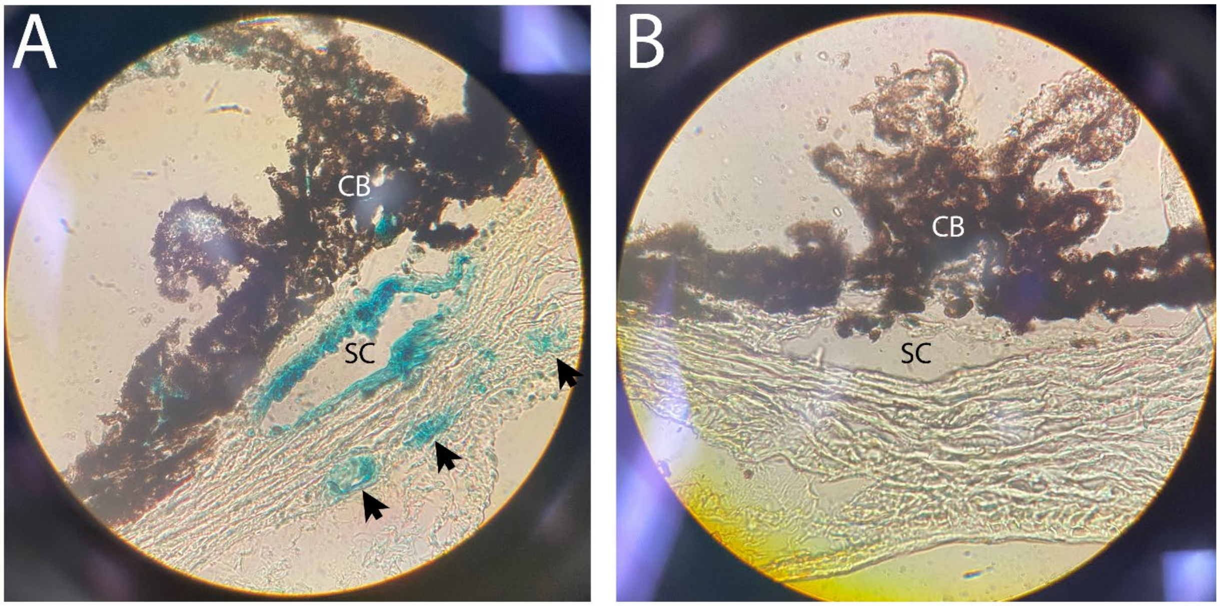

Supplemental Figure 1: Spontaneous Cre recombination observed in *R26R/Cre;Nos3^{flox}* mice using X-gal staining. (A) In pilot studies, Lac Z expression (blue) was unexpectedly present in endothelia of SC and DVs (arrows) of all *R26R/Cre;Nos3^{flox}* mice at 2-3M of age (n=12 eyes), whether they had received topical tamoxifen treatment or not. **(B)** X-gal staining was carried out on *R26R* and *C57BL/6J* mice (control) to ensure the stain was not showing false positive results. There was no Lac Z expression (blue) present in endothelial cells of SC or DVs in any of these mice (n=6 eyes).

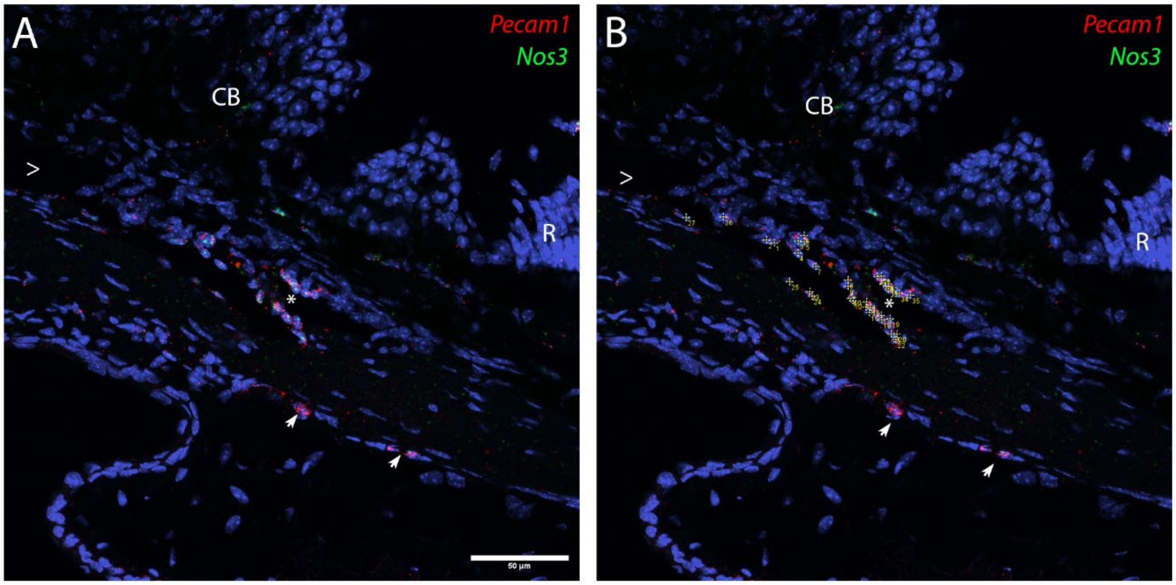

Supplemental Figure 2: Method used to quantify *Nos3* mRNA expression, using RNAscope technology. (A) A probe for *Pecam1* (red) was used to identify endothelial cells, a probe for *Nos3* (green) was used to determine mRNA expression of *Nos3* and DAPI (blue) labeled cells. **(B)** To quantify expression, fluorescent puncta corresponding to each individual *Nos3* mRNA molecule that overlapped with a cell (DAPI) along the SC lumen and distal vessels was counted. This was then expressed as percentage of puncta per area (circumferential length) and normalized to the expression of WT mice. Images shown here are reused from Figure 2B to demonstrate quantitation method. CB: ciliary body, >: open iridocorneal angle, R: retina, *: Schlemm's canal, and arrows: distal vasculature. Scale bar is 50 µm.

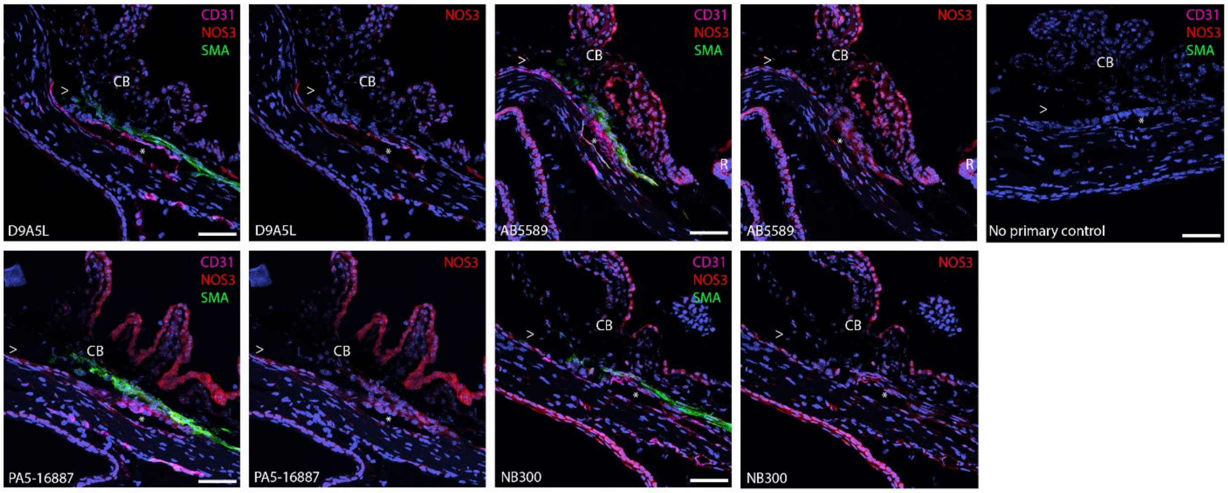

Supplemental Figure 3: NOS3 protein expression observed in Schlemm's canal and surrounding distal vasculature in *C57BL/6J* mice. Four different commercially available NOS3 antibodies were tested on 12 µm sections of anterior segment tissue from *C57BL/6J* mice at P90. These antibodies included Cell Signaling (D9A5L), Abcam (AB5589), Invitrogen (PA5-16887) and Novus (NB300). Abcam, Invitrogen and Novus were all found to be non-specific and therefore not suitable. Cell Signaling NOS3 antibody shows NOS3 protein expression (red) observed in Schlemm's canal (*) and surrounding distal vasculature in *C57BL/6J* mice at P90. Schlemm's canal and the surrounding distal vasculature are identified using CD31 staining (magenta) and co-localized with NOS3 (red). The TM, identified using αSMA (green) does not express NOS3. No primary antibody control has also been included for reference. DAPI is stained blue. Representative images above are the results of n=3 eyes (both males and females), 5-10 sections per eye. CB: ciliary body, >: open iridocorneal angle, *: Schlemm's canal, and arrows: distal vasculature. Scale bar is 50 µm.

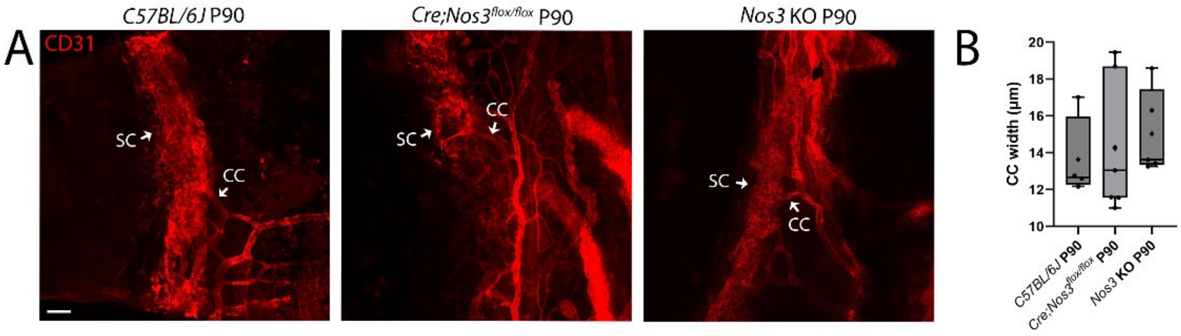

Supplemental Figure 4: No difference in collector channel diameter was observed between strains. **(A)** Representative confocal maximum projection images of limbal tissues from *C57BL/6J*, *Cre;Nos3$^{flox/flox}$* and *Nos3 KO* mice, all at P90. Whole mounts were labelled with endothelial cell marker CD31 (red), which shows the distal vasculature (Figure 4, main text), including collector channels (CC) and Schlemm's canal (SC). Scale bar represents 50 µm. **(B)** Analysis of collector channel width showed no significant difference in size between groups with mean values of 13.6 ± 2.3 µm for *C57BL/6J* mice (n=4 eyes), 14.2 ± 3.5 µm for *Cre;Nos3$^{flox/flox}$* mice (n=7 eyes) and 15.0 ± 2.3 µm for *Nos3 KO* mice (n=5 eyes, P>0.7 between all groups, one-way ANOVA with Tukey's multiple comparisons test). Each data point represents the average measurement taken from four quadrants of a single eye for each group.

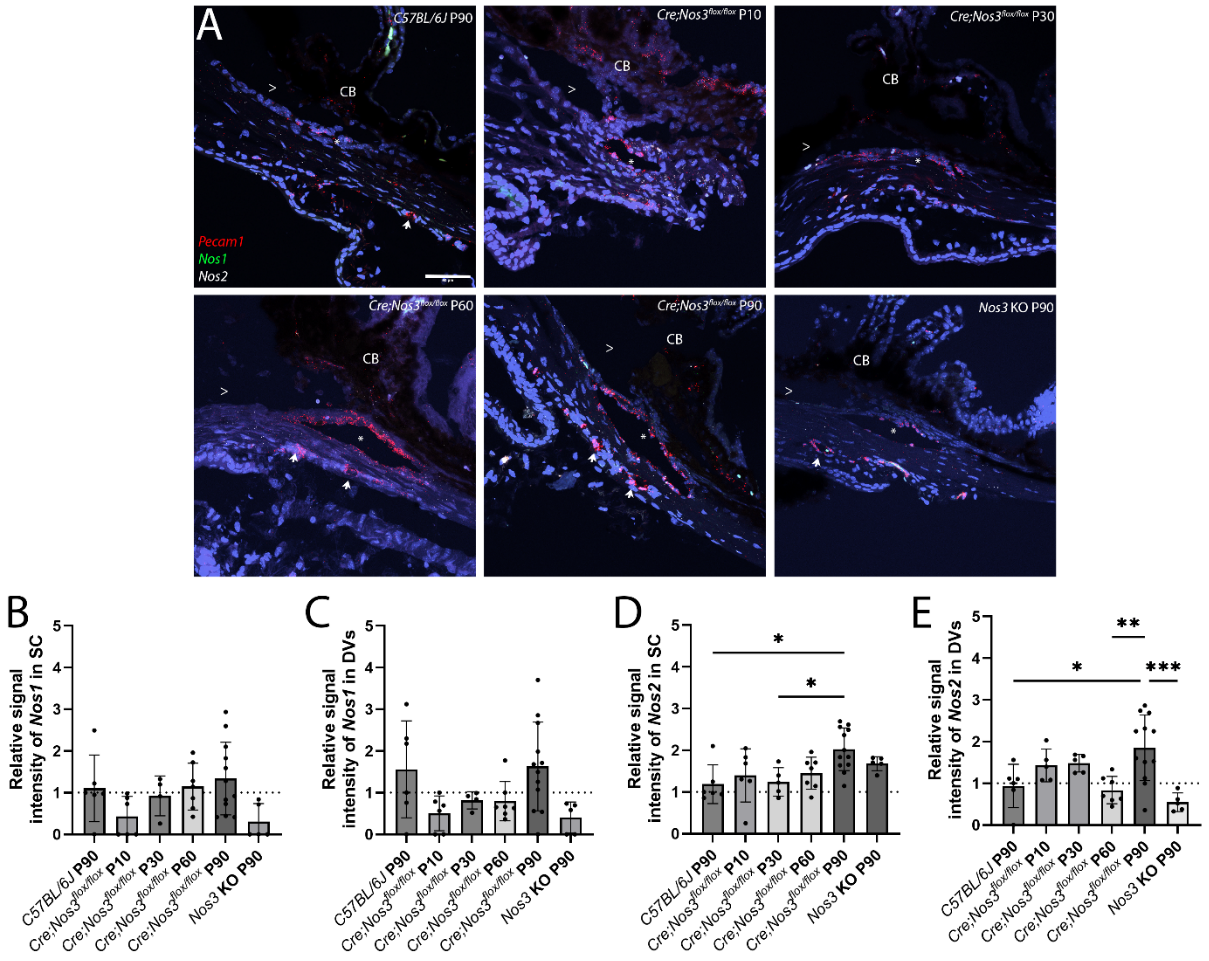

Supplemental Figure 5: RNAscope showing *Nos1* and *Nos2* mRNA expression. (A) Representative images of *Cre;Nos3*$^{flox/flox}$ mice P10-90, *C57BL/6J*, and *Nos3* KO control mice (both at P90) are shown above. *Pecam1* (red) was used to identify the SC and DV regions; *Nos1* (green) and *Nos2* (grey) are also shown. DAPI is stained in blue. CB: ciliary body, >: open iridocorneal angle, R: retina, *: Schlemm's canal, and arrows: distal vasculature. Scale bar is 50 µm. **(B)** Relative signal intensity of *Nos1* mRNA expression in the SC region showed no change in *Cre*;*Nos3*$^{flox/flox}$ mice P10-90 compared to *C57BL/6J* and *Nos3* KO control mice. **(C)** Relative signal intensity of *Nos1* mRNA expression in the DV region also showed no change in *Cre;Nos3*$^{flox/flox}$ mice P10-90 compared to *C57BL/6J* and KO control mice. **(D)** Relative signal intensity of *Nos2* mRNA expression in the SC region showed a significant increase in expression at P90 in *Cre*;*Nos3*$^{flox/flox}$ mice compared to *Cre*;*Nos3*$^{flox/flox}$ mice at P30 (P=0.0337) and *C57BL/6J* at P90 (P=0.0106). **(E)** Relative signal intensity of *Nos2* mRNA expression in the DV region showed a significant increase in expression in *Cre*;*Nos3*$^{flox/flox}$ mice at P90 compared to *Cre*;*Nos3*$^{flox/flox}$ mice at P60 (P=0.0044) and *C57BL/6J* (P=0.0197) and KO control mice (P=0.0008) both at P90. This RNAscope data supports IHC analysis for both NOS1 (Supplemental Figure 6) and NOS2 protein (Supplemental Figure 5). Each individual data point represents a single unpaired eye and statistical analysis was carried out using one-way ANOVA with Tukey's multiple comparisons test. *=P<0.05, **=P<0.01, ***=P<0.001 and ****=P<0.0001.

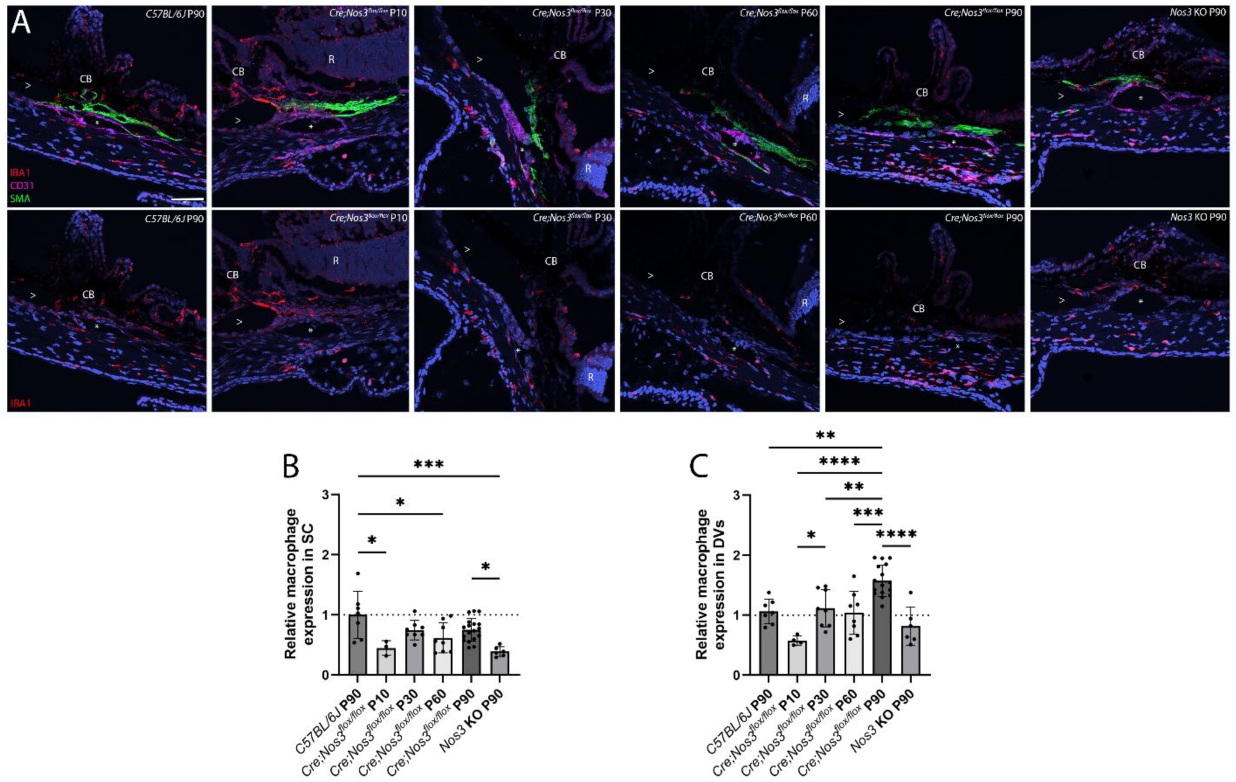

Supplemental Figure 6: Increased numbers of IBA1+ macrophages in the DV region of *Cre*;*Nos3*$^{flox/flox}$ mice at P90. (A) IBA1+ macrophages (red), alongside CD31 (magenta), identifying endothelial cells of SC and the distal vasculature, and αSMA (green) identifying the TM. Representative images of *Cre*;*Nos3*$^{flox/flox}$ mice P10-90 are shown above, alongside control *C57BL/6J* and KO mice. Image of *Cre*;*Nos3*$^{flox/flox}$ mouse from Figure 5D (P90) reappears here to show comparison to other ages. **(B)** Semi-quantification of IBA1+ macrophages in the SC region showed decreased macrophage expression in *Cre*;*Nos3*$^{flox/flox}$ mice P10-90 and KO mice. **(C)** Semi-quantification of IBA1+ macrophages near the DV region showed significant increase in the number of macrophages to this area in *Cre*;*Nos3*$^{flox/flox}$ mice at P90 compared to younger ages and both *C57BL/6J* and KO control mice. DAPI is stained in blue. CB: ciliary body, >: open iridocorneal angle, R: retina, *: Schlemm's canal, and arrows: distal vasculature. Scale bar is 50 μm. This figure has also been adapted for the main text to include represented images and analysis of *Cre*;*Nos3*$^{flox/flox}$, *C57BL/6J*, and KO mice only at P90. This allows for larger, clearer analysis to be included in the main text. Each individual data point represents a single unpaired eye and statistical analysis was carried out using one-way ANOVA with Tukey's multiple comparisons test. *=P<0.05, **=P<0.01, ***=P<0.001 and ****=P<0.0001.

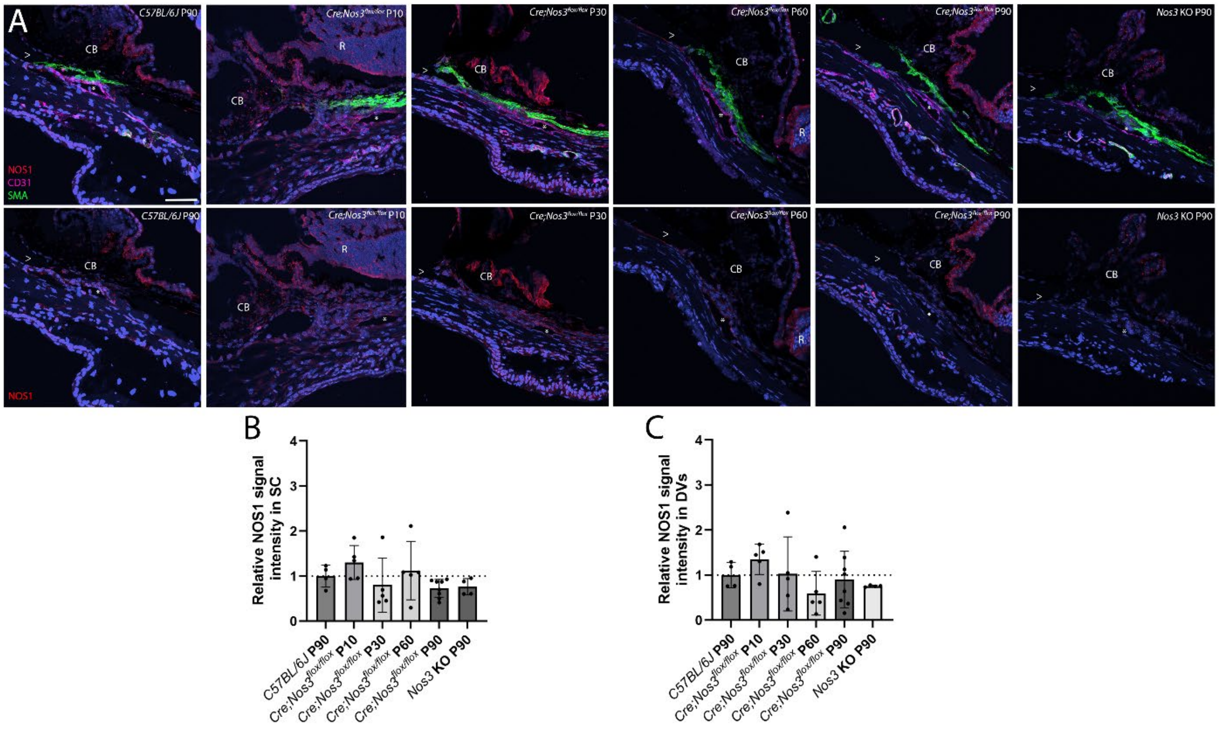

Supplemental Figure 7: NOS1 protein expression remained unchanged in both SC and DV region in *Cre*;*Nos3*$^{flox/flox}$ mice. (A) NOS1 (red) protein expression alongside CD31 (magenta) identifying endothelial cells of SC and the distal vasculature, and αSMA (green) identifying the TM. *Cre*;*Nos3*$^{flox/flox}$ mice P10-90 are shown in this figure, alongside C57BL/6J and KO control mice at P90. **(B)** Semi-quantification of NOS1 protein expression in the SC region showed no difference between ages of *Cre*;*Nos3*$^{flox/flox}$ mice compared to control mice. **(C)** Semi-quantification of NOS1 protein expression near the DVs also showed no difference between ages of *Cre*;*Nos3*$^{flox/flox}$ mice compared to control mice. DAPI is stained in blue. CB: ciliary body, >: open iridocorneal angle, R: retina, *: Schlemm's canal, and arrows: distal vasculature. Scale bar is 50 μm. Each individual data point represents a single unpaired eye and statistical analysis was carried out using one-way ANOVA with Tukey's multiple comparisons test.

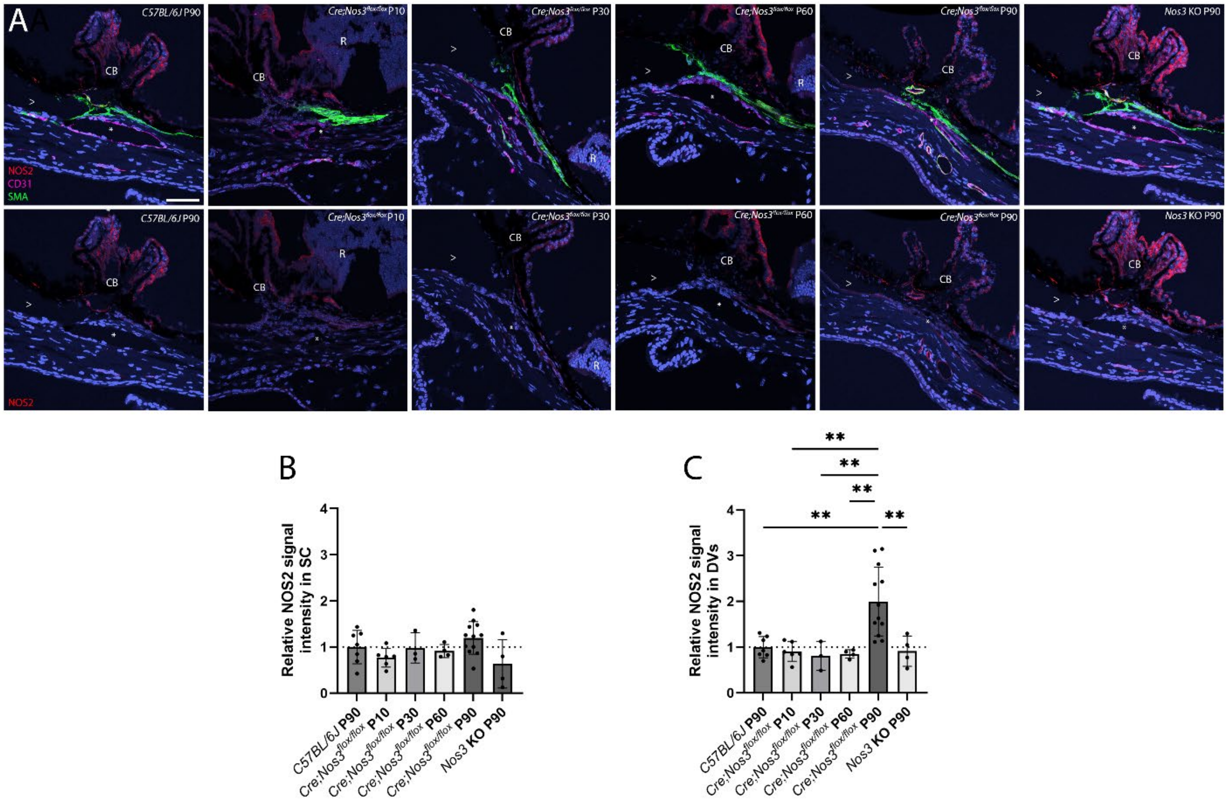

Supplemental Figure 8: Increased NOS2 protein expression observed in the DV region of *Cre*;*Nos3*$^{flox/flox}$ mice at P90. (A) NOS2 (red) protein expression alongside CD31 (magenta) identifying endothelial cells of SC and the distal vasculature, and αSMA (green) identifying the TM. Representative images of *Cre*;*Nos3*$^{flox/flox}$ mice P10-90 are shown above, alongside control *C57BL/6J* and KO mice. Images from Figure 5G (P90) were reused here to show comparison to other ages. **(B)** Semi-quantification of NOS2 protein expression in the SC region showed no difference in *Cre*;*Nos3*$^{flox/flox}$ mice overtime compared to control mice. **(C)** Semi-quantification of NOS2 protein expression near the DV region showed significant increase in NOS2 expression at P90 in *Cre*;*Nos3*$^{flox/flox}$ mice compared to younger ages and both *C57BL/6J* and KO mice. DAPI is stained in blue. CB: ciliary body, >: open iridocorneal angle, R: retina, * Schlemm's canal, and arrows: distal vasculature. Scale bar is 50 µm. This figure has been adapted for the main text to include represented images and analysis of *Cre*;*Nos3*$^{flox/flox}$, *C57BL/6J*, and KO mice only at P90. This allows for larger, clearer analysis to be included in the main text. Each individual data point represents a single unpaired eye and statistical analysis was carried out using one-way ANOVA with Tukey's multiple comparisons test. *=P<0.05, **=P<0.01, ***=P<0.001 and ****=P<0.0001.

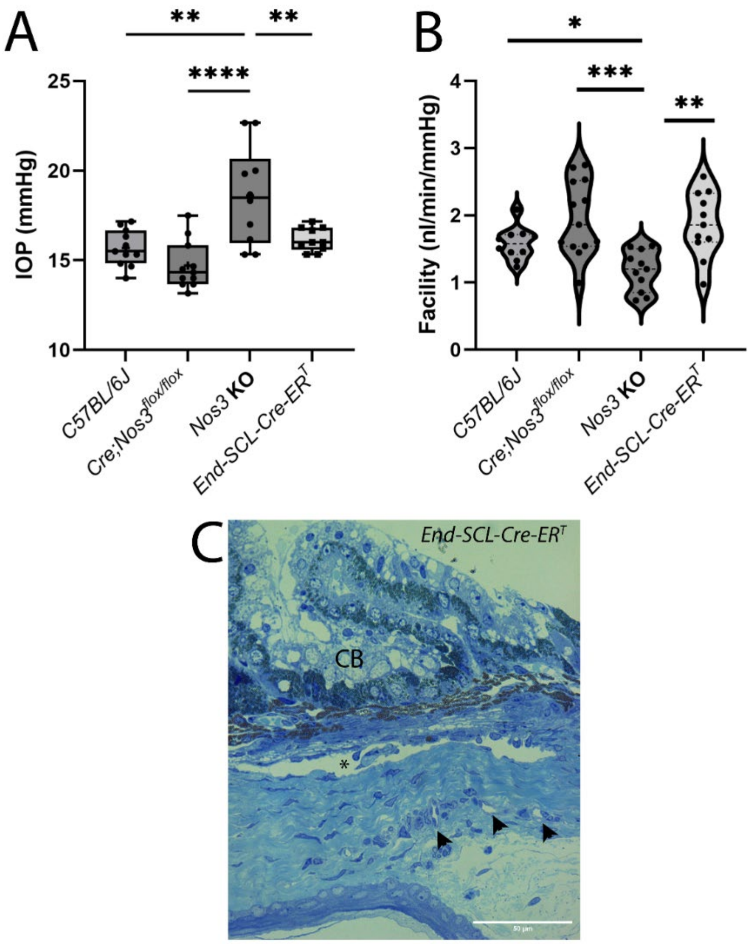

Supplemental Figure 9: Characterization of *End-SCL-Cre ER*^{T} mice. The genetic background of the *Cre*;*Nos3*$^{flox/flox}$ mice included expression of the Cre-ER^{T} fusion protein driven by a 5'-endothelial-SCL enhancer element (*End-SCL-Cre ER*^{T}). To ensure IOP and facility results observed in *Cre*;*Nos3*$^{flox/flox}$ mice were the result of *Nos3* being excised and not the effects of Cre-ER^{T} expression, we purchased *End-SCL-Cre ER*^{T} mice at 10-12 weeks old (n=3 males and n=3 females) from The Jackson Laboratory (Strain #; 037467). **(A)** A mean IOP value of 16.2 ± 0.7 mmHg was observed in these *end-SCL-Cre-ER*^{T} mice, which was significantly different from *Nos3* KO mice at 18.6 ± 2.7 mmHg (P=0.0088) but not from *Cre*;*Nos3*$^{flox/flox}$ mice (P=0.1825) or C57Bl/6J mice (P=0.9087). **(B)** A mean facility value of 1.8 [1.5, 2.2] nl/min/mmHg (geometric mean [95% CIs]) was observed in these *end-SCL-Cre-ER*^{T} mice, which was significantly different from *Nos3* KO mice at 1.1 [0.8, 1.4] nl/min/mmHg (P=0.0012) but not from C57BL/6J (P=0.6213) or *Cre*;*Nos3*$^{flox/flox}$ mice (P=0.9788). **(C)** Semi-thin blue staining was also carried out on tissue sections from these mice and no obvious gross morphological differences were observed in these mice compared to all other strains. Representative image at 63X is shown above for *End-SCL-Cre ER*^{T} mice at 10-12 weeks. CB: ciliary body, *: Schlemm's canal, and arrows: distal vasculature. 50 μm scale bar is shown. This further supports our hypothesis that results observed in *Cre*;*Nos3*$^{flox/flox}$ mice are the results of postnatal deletion of the *Nos3* gene. Each individual data point represents a single unpaired eye and statistical analysis was carried out using one-way ANOVA with Tukey's multiple comparisons test. *=P<0.05, **=P<0.01, ***=P<0.001 and ****=P<0.0001.

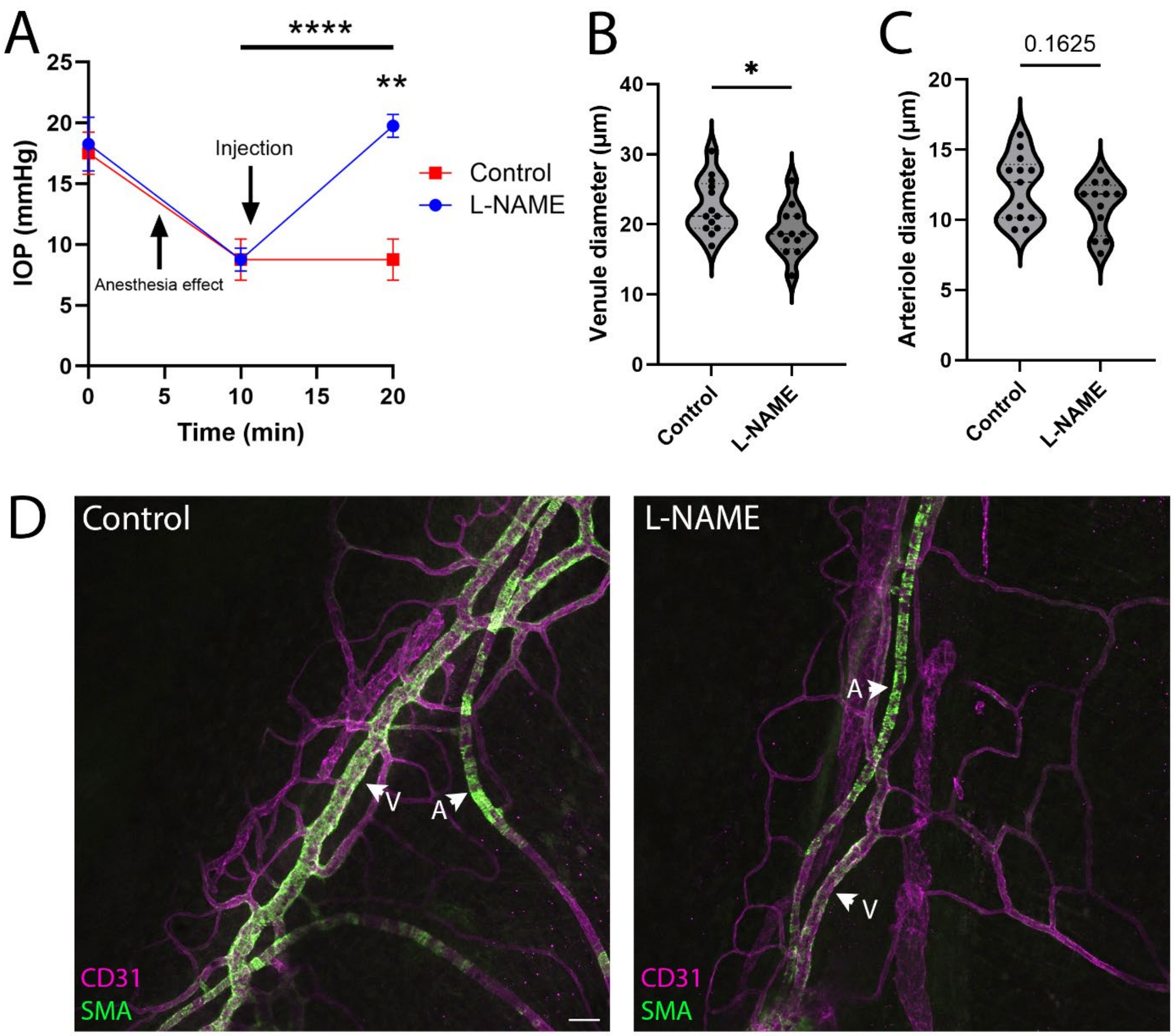

Supplemental Figure 10: Elevated IOP and narrowing of distal vessels was observed following subconjunctival treatment with L-NAME in *C57BL/6J* mice. (A) IOP measurements were taken pre injection and 10 min post anesthesia, following bottoming out of IOP due to ketamine/xylazine anesthesia. There was no difference in IOP in control eyes (red) following a sham injection of PBS. In contrast, L-NAME treated eyes had a significant increase in IOP 10 min post injection (P=0.0016). Distal vessel characterization was then carried out and vessel diameter measured. **(B)** A significant decrease in venule diameter was observed in L-NAME treated eyes compared to control eyes (P=0.0262). **(C)** No significant difference in arteriole diameter was observed between control and L-NAME treated eyes. As well, there was no difference in capillary diameter (data not shown). Each data point represents a single measurement from each quadrant of n=3 eyes. Unpaired analysis was carried out on this data due to the small sample size. Statistical differences was indicated as *=P<0.05, **=P<0.01 and ****=P<0.0001. **(D)** Representative images showing venules (V) and arterioles (A) of the distal vasculature in control and L-NAME treated eyes of *C57BL/6J* mice at P90. The distal vasculature was stained with both endothelia marker CD31 (magenta) and smooth muscle actin (SMA) marker (green). Scale bar represents 50 µm.

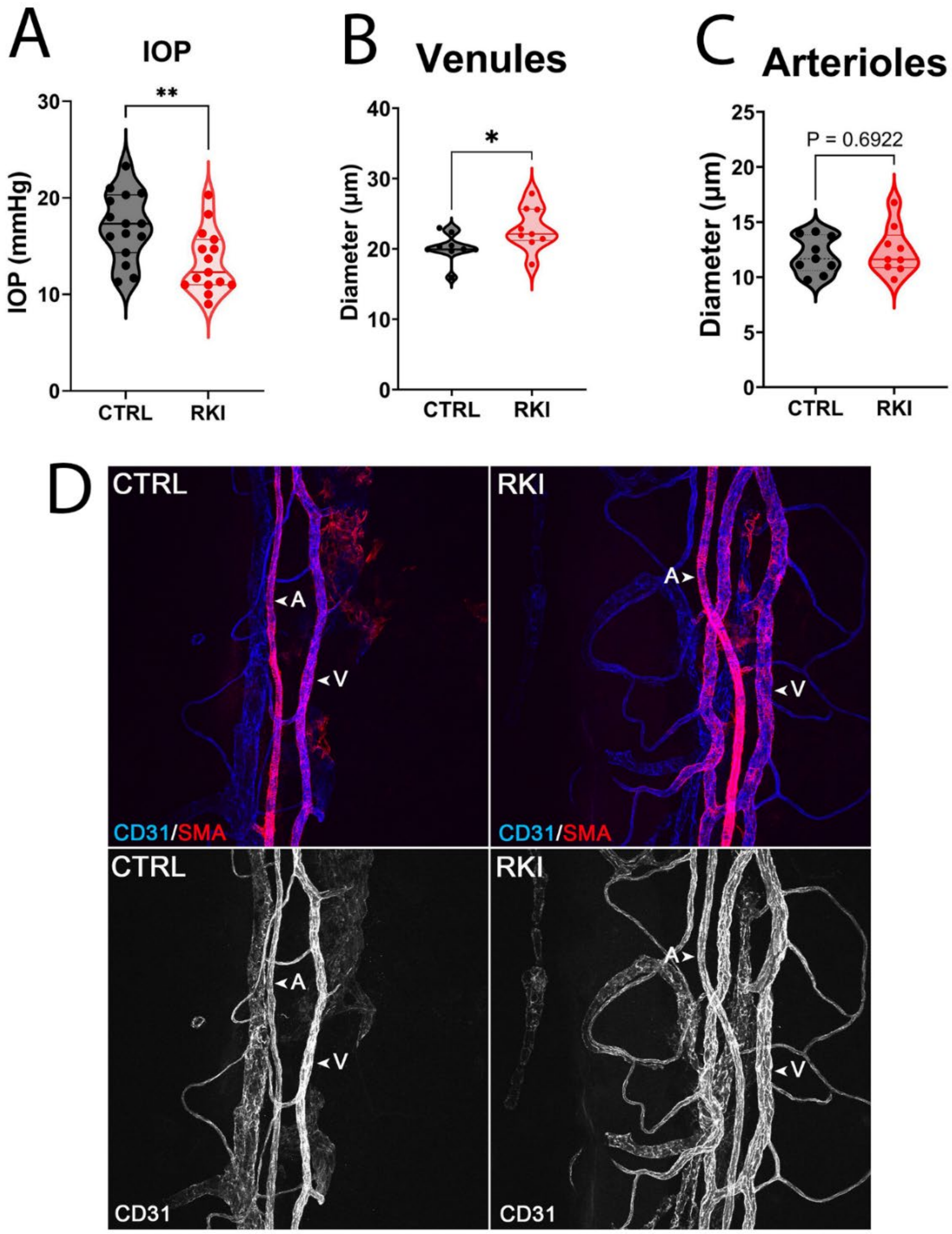

Supplemental Figure 11: A rho kinase inhibitor lowered IOP and increased distal venule diameter following immersion fixation: (A) IOP measurements were taken 2 hr after topical treatment with either Rhopressa®, a rho kinase inhibitor (RKI), or control (CTRL). RKI treated eyes had significantly lower IOPs following a single drop of the RKI. Immediately after IOP measurements, eyes were enucleated, fixed by immersion, and DV staining with CD31 and SMA markers, both venule and arteriole diameter for each eye was measured. **(B)** A significant increase in the diameter of the RKI treated venules was observed compared to control eyes. **(C)** No significant difference in arteriole diameter was observed between control and RKI treated eyes. Each data point represents a single unpaired eye, with statistical differences indicated as *=P<0.05 and **=P<0.01. **(D)** Representative images showing venules (V) and arterioles (A) of the distal vasculature in *C57BL/6J* mice stained with endothelia marker CD31 (blue) and smooth muscle actin (SMA) marker (red). Images showing CD31 alone in grey are below.

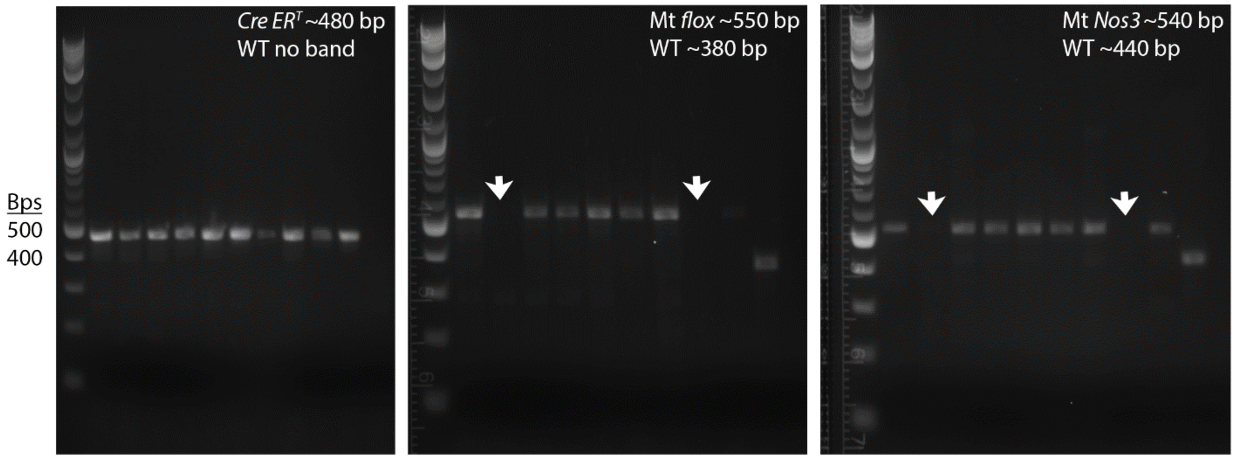

Supplemental Figure 12: Representative images of genotyping carried out for all *Cre*;*Nos3*$^{flox/flox}$ mice. Genotyping was carried out using toe cuts of all *Cre*;*Nos3*$^{flox/flox}$ pups aged P7-10. Genotyping was carried out for *Cre-ER*^{T}, *flox/flox,* and *Nos3*, separately. Mice expressing *Cre-ER*^{T} have a band at ~480 bp, while mice not expressing *Cre-ER*^{T} or WT mice have no band. Mice expressing *flox/flox* have a band at ~550 bp and WT mice have a band at ~380. Mice expressing *Nos3* have a band at ~540 bp and WT mice at ~440 bp. All *Cre*;*Nos3*$^{flox/flox}$ mice used in this study expressed *Cre-ER*^{T}/*flox/flox*/*Nos3*. All mice genotyped expressed *Cre-ER*^{T}. Some mice were missing elements (arrows) including *flox/flox* and *Nos3*. These mice were not included in the study.

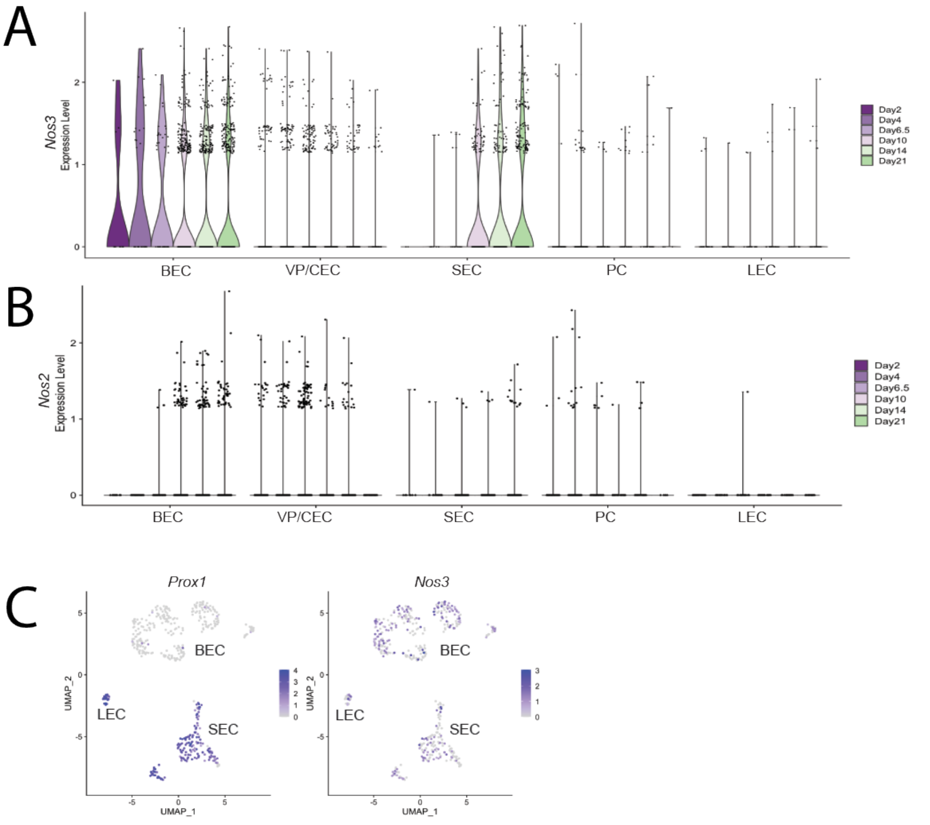

For figure legend:

BEC : Blood endothelial cells
VP/CEC : Vascular progenitors/
Collector channel endothelial cells
SEC : Schlemm's canal endothelial cells
PC : Progenitor cells
LEC : Lymphatic endothelial cells

Supplemental Figure 13: Single cell data of both *Nos3* and *Nos2* in limbal tissue of *C57BL/6J* mice through development. Single cell RNA-seq of *C57BL/6J* limbal tissue through development (Postnatal day 2.5-21) identifies various expression levels of both *Nos3* and *Nos2* in different endothelial cell types. This helped us identify non-specific staining of antibodies. **(A)** Violin plot showing *Nos3* expression of *C57BL/6J* limbal tissue through development (Day 2.5-21). *Nos3* begins to be expressed around Day 4-6.5 in Schlemm's canal endothelial cells (SEC) of *C57BL/6J* mice. These cells are in an immature state at this time. *Nos3* becomes more highly expressed throughout development at Day 10-21 in SECs and blood endothelial cells (BEC). This was confirmed in our RNAscope and IHC staining (Figure 2) with *Nos3*/NOS3 expression abundant along endothelial cells of SC and the DVs. **(B)** *Nos2* is not highly expressed in SECs at Day 10-21 in *C57BL/6J* mice, unlike *Nos3*. *Nos2* expression is more confined to BECs (Day 10-21) and vascular progenitor cells (VP) (Day 2-14). We observed this in our RNAscope (Supplemental Figure 4) and IHC staining (Figure 5) and therefore, makes the phenomenon observed in *Cre*;*Nos3*$^{flox/flox}$ mice all the more interesting. **(C)** Plot showing both *Prox1* and *Nos3* expression in 90 day old *C57BL/6J* mice. *Prox1* is highly expressed in LEC and SEC, while a few cells are found in BEC. *Nos3* however, is expressed in BEC, LEC and SEC. We observed *Nos3* mRNA and NOS3 protein in all three of these cell types in both SC endothelial cells and the distal vasculature (Figure 2).

	C57BL/6J		*Cre;Nos3$^{flox/flox}$*		*Nos3* KO	
	Male (n=19)	Female (n=18)	Male (n=18)	Female (n=20)	Male (n=17)	Female (n=14)
Weight (g)	26.1 ± 1.9 **	20.8 ± 1.9 **	26.7 ± 2.6 ***	22.1 ± 1.5 ****	23.8 ± 1.3	18.6 ± 1.2
Litter size		5.8 ± 1.6 ***		5.2 ± 1.7 ***		3.0 ± 1.2

Supplemental Table 1. Mean weight (g) and litter size of *C57BL/6J*, *Cre*;*Nos3*$^{flox/flox}$, and *Nos3* KO mice. The mean weight (g) of both male and female mice (separate groups) was taken for all mice at P90. It has previously been reported that global *Nos3* KO mice have developmental defects and may have lower body weights than healthy control animals (1). Using *C57BL/6J* mice as a control group, both males and females had significantly increased body weights compared to global KO mice (26.1 ± 1.9 and 20.8 ± 1.9 versus 23.8 ± 1.3 and 18.6 ± 1.2, respectively, P=0.0023 and P=0.0081, respectively, one-way ANOVA with Tukey's multiple comparisons test). *Cre*;*Nos3*$^{flox/flox}$ males and females also had significantly increased body weight compared to global KO mice (26.7 ± 2.6 and 22.1 ± 1.5 versus 23.8 ± 1.3 and 18.6 ± 1.2, respectively, P=0.0001 and P=0<0.001, respectively). There was no difference in body weight in both males and females between *C57BL/6J* mice and *Cre*;*Nos3*$^{flox/flox}$ mice (26.1 ± 1.9 and 20.8 ± 1.9 versus 26.7 ± 2.6 and 22.1 ± 1.5, respectively, P=0.9572 and P=0.3056, respectively. Fertility issues have also been reported in global KO mice, therefore mean litter sizes were calculated for all. A significant increase in the average litter size was reported in both *C57BL/6J* mice (5.8 ± 1.6, n=12, P=0.0001) and *Cre*;*Nos3*$^{flox/flox}$ mice (5.2 ± 1.7, n=17, P=0.0009) compared to global *Nos3* KO mice (3.0 ± 1.2, n=14). There was no significant difference in litter size between *C57BL/6J* mice and *Cre*;*Nos3*$^{flox/flox}$ mice (5.8 ± 1.6 versus 5.2 ± 1.7, P=0.5817).

Supplemental Methods

Animals

Three breeding pairs of endothelial-specific tamoxifen inducible Cre-ER^{T} mice, targeting *Nos3* (*Endo-SclCre-ER*^{T}*;Nos3*$^{flox/flox}$) (2) were sent from Vanderbilt University to Duke University, which we used to build our colony. All studies involving animals were compliant with the ARVO Statement for the User of Animals in Ophthalmic and Vision Research and the National Institutes of Health Guide for the Care and Use of Laboratory Animals. *Cre;Nos3*$^{flox/flox}$ mice were initially bred with *R26R* reporter mice from The Jackson Laboratory (stock #: 003474) to give *R26R/Cre;Nos3*^{flox} mice. *R26R/Cre;Nos3*^{flox} mice were used to characterize Cre-ER^{T} expression, using X-gal staining. Due to the spontaneous Cre-ER^{T} recombination observed in all *R26R/Cre;Nos3*^{flox} mice, there were no littermate controls for this study. *Cre;Nos3*$^{flox/flox}$ mice were also crossed with a second reporter strain mouse, *Ai9* tdTomato (The Jackson Laboratory, strain #007909) to confirm the spontaneous Cre recombination observed in *R26R/Cre;Nos3*^{flox} mice. *Cre;Nos3*$^{flox/flox}$ mice were bred on a *C57BL/6J* background, however and so *C57BL/6J* mice were used as control animals. *Nos3* global knockout mice (*Nos3* KO) purchased from The Jackson Laboratory (stock #: 002684) were also used as control animals. These *Cre;Nos3*$^{flox/flox}$ mice, received from Dr. Takahashi's lab were on a genetic background with endothelial-SCL-Cre-ER^{T} expressing Cre-ER^{T} fusion protein driven by 5'-endothelial-stem cell leukemia (Scl) promoter and enhancer element. Therefore, we also purchased *end-SCL-Cre-ER*^{T} mice from The Jackson Laboratory (stock #: 037467). We used these animals as control mice to ensure the effects on IOP and facility were not due to Cre toxicity. Genotyping was performed using toe cuts taken from pups aged P7-P10. The toes were digested using Terra PCR Direct Genotyping Kit (Takara Bio). PCR was carried out for *Cre-ER*^{T}, *Nos3,* and *flox/flox*, separately. All primers were purchased from IDT and listed below. An example of the genotyping carried out is in Supplemental Figure 12.

Tamoxifen preparation and use

Tamoxifen was prepared into 20 mg/ml stock solution. Firstly, corn oil (Sigma) was warmed to 37°C and tamoxifen powder (Sigma-Aldrich) added. This solution was incubated at 37°C for 30 min, protected from

light, then vortexed for 1-2 hr until mixed properly. The solution must be warmed throughout this process in order to make a homogenous solution. Aliquots can then be stored at -20°C but must be warmed to room temperature before use. This tamoxifen solution was then pipetted (10 µl drop) directly onto the mouse eye three times daily for 3 days. The mice were then left for 48 hr until eyes were enucleated and X-gal stained for Cre-ER^T expression. Control mice received corn oil with no tamoxifen added.

X-gal staining

Lac Z expression was detected using X-gal staining (3, 4). This was carried out using an X-Gal staining kit (OZ Biosciences). Briefly, *Cre;Nos3$^{flox/flox}$* were crossed with *R26R* mice (stock #: 003474) from The Jackson Laboratory. The offspring of these mice (*R26R/Cre;Nos3^{flox}*) should have the neo cassette removed by Cre-ER^T recombination, resulting in Lac Z expression in Cre-expressing tissues. Eyes were enucleated and placed in fixing buffer (formaldehyde, glutaraldehyde, PBS) overnight at 4°C. Eyes were then washed twice with 1X PBS to remove fixative. Eyes were dissected to remove the back of the eye, cutting below the limbus to keep anterior segment outflow tissue intact. The lens was then removed and gently, using a thin paint brush, teased the iris away from the inside of the cornea to allow X-Gal stain to easily enter tissues of the outflow pathway. Eyes were then incubated with freshly prepared 1X staining solution (potassium ferricyanide, potassium ferrocyanide Magnesium Chloride in PBS) in 1X X-Gal solution (5-bromo-4-chloro-3-indoyl-β-D-Galactopyranoside in DMF) overnight at 37°C. The X-Gal staining solution was then removed and eyes were washed with 1X PBS. Eyes were places in 30% sucrose overnight at 4°C, until the eye cup had fallen to the bottom of the tube. Eye cups were then placed in Tissue-Tek OCT mounting media (Sakura) in a 10x10x5 mm cryomold biopsy and placed in the -80°C freezer. These blocks were placed in a sealed container until ready to section. 12 µm sections were cut using a cryostat and placed on positively charged slides. These slides were stored at -20°C until ready to use. A hydrophobic barrier pen (Vector ImmEdge Hydrophobic Barrier Pen) was used to create a water barrier around the sections. Slides were washed carefully two times with 1X PBS, adding dropwise to remove OCT. Slides were then mounted and coverslip added. Lac Z staining was imaged at both 20X and 63X magnification using a Zeiss

Axio Microscope (Carl Zeiss Microscopy). *R26R* and *C57BL/6J* mice with Lac Z staining were used as negative controls. Two trained, masked observers scored the images based on a scoring system from 0-3 with example images (no stain=0, very light staining=1, darker staining=2, and very intense staining=3). Each observer was given an example image to represent each score. The results are an average of the two masked observers' scores for each time point and shown in Figure 1. Unlike IHC, X-gal staining was carried out using light microscopy, which was converted to gray scale. Due to pigment in and around the SC/TM region, we were unable to use the semi-quantitative approach used for IHC.

Immunohistochemistry

Eyes were enucleated and fixed with 4% PFA overnight at 4°C. PFA was removed and eyes were washed twice with 1X PBS. The back of the eye was removed, cutting below the limbus, and the lens was removed. The anterior segment was placed in 30% sucrose overnight at 4°C. The anterior cup was carefully placed in OCT in a 10x10x5 mm cryomold biopsy and put in the -80°C freezer. These blocks were then placed in a sealed container until ready to section. 12 μm sections were cut using a cryostat and placed on positively charged slides. These slides were stored at -20°C until ready to use. A hydrophobic barrier pen was used to create a water barrier around the sections. Slides were washed carefully two times with 1X PBS, adding dropwise to remove OCT. Slides were then washed for 2 min with 70% Ethanol and 2 min with 100% Ethanol. The tissue was blocked with 10% goat serum in 1% Triton-X solution for 1 hr at room temperature. The tissue was then incubated with various primary antibodies such as: CD31 at 1:50 (Millipore, mAb13982 [2H8]), αSMA conjugated to Alexa 488 at 1:200 (Invitrogen, #53-9760-82 [1A4]), NOS1 at 1:800 (Abcam, ab76067 [EP1855Y]), NOS2 at 1:100 (Cell Signaling, #13120 [D6B6S]), IBA1 at 1:200 (Wako, #019-19741), ELAM1 at 1:100 (BD Biosciences, #550290 [CD62E]) and NOS3 at 1:100 (Cell Signaling, mAb#32027 [D9A5L]) in blocking buffer overnight at 4°C. Various other NOS3 antibodies were trialed, including Novus (NB300), Abcam (Ab5589), and Invitrogen (PA5-16887) and were found to be non-specific and therefore not suitable (Supplemental Figure 3). Tissue was then washed three times for 5 min with 1X PBS. Tissue was incubated with secondary antibody goat anti-Armenian Hamster AI647 (Jackson

ImmunoResearch Laboratories Inc., #127-605-160) and goat anti-Rabbit AI594 (Jackson ImmunoResearch Laboratories Inc., #111-585-144), both at 1:200 in 1X PBS for 1 hr at room temperature. Samples were washed three times for 5 min with 1X PBS and mounted using ProLong Diamond Antifade Mountant with DAPI (Thermo Fisher Scientific) and a coverslip added. Slides were left to dry overnight at room temperature before sealing with clear nail polish and imaging. A negative control sample was always prepared with no primary antibody added, just secondary antibodies. Images were taken using a Nikon Eclipse 90i confocal laser-scanning microscope (Melville). Z-stack images of 1 µm thickness were taken of each section and converted into maximum projection images that were used for analysis.

Semi-quantitative analysis of immunohistochemistry

Using Image J (FIJI) Software (National Institute of Health (NIH)), CD31 (magenta) staining on maximum projection images was used to identify Schlemm's canal (Supplemental Figure 3) and the surrounding distal vasculature. Using the polygon selection tool, the area of interest (Schlemm's canal lumen, including inner and outer wall and distal vessels, separately) was then highlighted. Using the channel manager (Shift + Z), the channel of interest (NOS1/NOS2/NOS3/IBA1/ELAM1) was then selected and saved (T). The results were then displayed (press M) including area, mean, min, max, IntDen and RawIntDen. Corrected total cellular fluorescence (CTCF) = IntDen – (Area selected x mean fluorescence of background) was then calculated in Excel. This was followed by calculation of fold change vs average *C57BL/6J* positive control sample. The area of the region of interest was important as the size of SC lumen changes with development and age of the mouse eye. It was essential to account for this when calculating relative protein expression for all markers.

RNAscope®

RNAscope® was carried out using RNAscope® Multiplex Fluorescent Reagent Kit v2-Mm (Advanced Cell Diagnostics a Bio Techne Brand). Probes for mouse *Nos3* (#443061), *Pecam1* (#316721-C2), *Nos1* (#437651-C3), and *Nos2* (#319131) were all purchased from ACD Bio. Fluorescent dyes Opal 690 (FP1497001KT), Opal 620 (FP1495001KT), and Opal 520 (FP1487001KT) (Akoya Biosciences) were

used. Briefly, eyes were enucleated and fixed in 4% PFA (made fresh) for 24 hr at 4°C. Dissect off the back of the eye, remove the lens, and place the anterior cup in 30% sucrose overnight at 4°C. Tissue was cryopreserved in OCT and placed at -80°C. Sections were cut at 10-15 μm thickness using SuperFrost Plus slides (Fisher Scientific). Slides were air dried at -20°C for 1-2 hr before moving to -80°C for storage until ready to use. RNAscope® was then carried out over two days as per manufacturer's protocol. Briefly, slides were initially baked for 30 min at 60°C in ACD hybridization oven. Slides were washed with PBS and dehydrated in a battery of Ethanol washes. A hydrophobic barrier was drawn around the sections and allowed to dry. The hydrogen peroxide incubation was carried out for 10 min at RT. The slides were post fixed for 10 min in 4% PFA at RT, for better attachment of tissue. The antigen retrieval step was then carried out on slides for 9 min at 95°C. The slides were rinsed with deionized water and then 100% Ethanol. Protease III was added to the slides and incubated for 30 min at 40°C in the hybridization oven. Probes were pre-warmed in oven for 10 min. The slides were washed again with deionized water. The probe mix was added to slides, in addition to positive and negative control slides, and placed in oven at 40°C for 2 hr. Slides were washed twice with 1X wash buffer at RT and placed in 5X SSC overnight at RT. The following day, slides were washed twice again with 1X wash buffer. AMP1 was added and placed in oven at 40°C for 30 min. Slides were washed twice in 1X wash buffer. This step was repeated for AMP2 and AMP3. HRP-C1 was then added and placed in oven at 40°C for 15 min. Slides were washed as before, and Opal 690 dye at 1:1000 dilution (made up fresh each time) was added to slides and placed in oven at 40°C for 30 min. Slides were washed as before, HRP-blocker added and placed in oven at 40°C for 15 min, and the slides were washed again. This step was repeated for HRP-C2 and HRP-C3. Opal dye 570 was used for HRP-C2, and Opal dye 520 was used if there was a third channel. DAPI was then added to the slides, which were mounted with Prolong Gold Antifade Mountant and left to dry overnight at RT. The slides can be stored for up to 2 weeks at 4°C before imaging. Images were taken using a Nikon Eclipse 90i confocal laser-scanning microscope (Melville). Z-stack images of 1 μm thickness, using the 40X objective lens, were taken of each section and converted into maximum projection images that were used for analysis.

Quantification of RNAscope®

All images had *Pecam1* to identify SC endothelial cells and DAPI (blue) to identify individual cells. Each individual dot for *Nos1* , *Nos2* and *Nos3* was counted, using the multipoint tool on ImageJ. A dot was counted if it was in a DAPI stained cell along SC lumen (identified with *Pecam1*). Dots were not counted outside of SC lumen or dots not on DAPI stained cells. The size of SC lumen (circumferential length), using the polygon selection tool on ImageJ, was also measured for each image. This accounted for changes in SC size as SC develops fully in the postnatal mouse. The number of dots counted was then divided by the length of SC lumen (region of interest) and calculated as percentage dots per area. This was then normalized for each mouse to average expression in *C57BL/6J* mice (Supplemental Figure 2).

Quantitative Real-Time PCR

Eyes were enucleated and a ring of outflow tissue was cut above and below the limbus, removing corneal tissue. Outflow tissues were pooled from both eyes of the same mouse. Lung tissue was also removed from the mice. Tissue was then homogenized in an RNase free tube, using an RNase free plastic pestle, in lysis buffer containing β-marcaptoethanol. RNA was extracted following manufacturer's protocol, using the Qiagen (Hilden, Germany) RNeasy kit. cDNA synthesis was then carried out, following manufacturer's protocol, using Maxima 1st strand synthesis kit (ThermoFisher, Waltham, MA). Real time PCR was then carried out using Taqman Fast Advance Master Mix (ThermoFisher, Waltham, MA). 500 ng of RNA was used for each sample, using dH_2O to dilute samples accordingly. Both *Nos3* (Mm00435217) and *β-actin* (Mm02619580) Taqman primers were used. Both a negative primer control and genomic DNA control were also included on the 96 well plate.

IOP measurements

Mice were placed in an anesthetic chamber and anesthetized by 2-3 minutes of exposure to 3% isoflurane and 1 L/min oxygen. Mice were then placed on a soft clay mold supported on an adjustable laboratory jack stand using a Bain coaxial circuit to maintain anesthesia with 1% isoflurane. The rebound tonometer (TonoLab; Icare) was mounted on a micromanipulator supported by a ring stand (to provide stability) with

the tip positioned approximately 2 mm from the central cornea along the optical axis. IOP was measured with 3 sets of 6 measurements with the data averaged, according to manufacturer's instructions (5, 6). Due to anesthesia reducing IOP, we fixed the IOP measurements to start 5 minutes after the onset of anesthesia, which appears to minimize the variation in IOP between individuals according to our previous studies. After the IOP measurement, the animal was allowed to recover in an incubator at 33°C for several minutes under supervision, before being returned to housing. IOP measurements were all carried out at the same time of day (10-12 PM).

Ex vivo measurement of conventional outflow facility

Conventional outflow facility measurements were carried out using the iPerfusion system, as previously described (7). Mice were euthanized by isoflurane inhalation, followed by decapitation. Eyes were carefully enucleated and immediately perfused. Briefly, eyes were mounted onto a platform in the perfusion chamber holder using a small amount of cyanoacrylate glue (Loctite). These chamber holders were filled with pre-warmed, degased D-glucose in ++ Ca/Mg phosphate-buffered saline (DBG, 5.5 mM) and kept at a temperature of 35°C. A sharpened, beveled glass microneedle, filled with DBG and connected to the perfusion system, was inserted into the anterior chamber using a micromanipulator, visualized under a stereomicroscope. The tubing was flicked to remove any anterior chamber deepening for all eyes. After a 1 hr acclimation at 12 mmHg, facility was measured over a sequential range of pressure steps, starting at 5 mmHg, increasing 1.5 mmHg each step until 17 mmHg, then decreasing to a final step at 8 mmHg. Data analysis was carried out using the iPerfusion software and MATLAB, as previously described (7, 8). A non-linear flow-pressure model was used to account for pressure dependence of outflow facility in mice. Statistical analysis of facility is taking into account the log-normal distribution of the data and the results are reported as geometrical mean [95% CIs].

Morphological analyses

Eyes were enucleated and fixed in 4% PFA overnight at 4°C. Eyes were then washed twice with 1X PBS. The posterior eye cup was removed, along with the lens, leaving the anterior segment. The anterior segment

was then cut into four quadrants. For gross morphology studies, two of these quadrants were used. Each sample was embedded in Epon, and 0.5 µm semi thin sections were cut, stained with 1% of methylene blue and examined by light microscopy (Axioplan2, Carl Zeiss MicroImaging). Visualized by light microscopy in semi-thin sections, the circumferential length of SC was measured and the number of GVs present along the inner wall of SC was counted by a trained masked observer. The length of SC was measured using the freehand-line tool on ImageJ, measuring from the anterior to posterior extremity of the IW of SC. The length of SC in each quadrant was then combined to derive the average for each eye. The average number of GVs in each SC were counted manually for each quadrant and averaged for each eye. A GV was identified as a large, unshaded outpouching of the IW of SC into its lumen.

OCT imaging

For OCT experiments, mice were anesthetized systemically by intraperitoneal injection of ketamine (100 mg/kg) and xylazine (10 mg/kg). Each mouse was then placed on a custom-made OCT imaging mount and in vivo imaging was performed using an Envisu R2200 Ultra-high-resolution SD-OCT system (Bioptigen, Inc., Research Triangle Park, NC). The 840-nm SD-OCT system used a customized 180-nm Superlum Broadlighter source (Carrigtwohill, County Cork, Ireland) providing 2-µm axial resolution, and a 12-mm telecentric lens bore for anterior segment OCT imaging (9). The OCT probe was aimed at the inferior lateral limbus and the image was centered and focused around the iridocorneal angle for each eye of each mouse.

Distal vessel characterization

Eyes were enucleated and fixed in 4% PFA overnight at 4°C. Eyes were then washed twice with 1X PBS. The posterior eye cup was removed, along with the lens, leaving the anterior segment. Relief cuts were made along each quadrant of the anterior segment. Using a narrow paint brush, the iris was teased away. Anterior segment tissue was then blocked in 5% goat serum in 0.1% Triton X in PBS for 1 hr at room temperature on a rocker. Tissue was then incubated with CD31 (Millipore, #Mab13982, [2H8]) and αSMA conjugated to Alexa 488 (Invitrogen, #53-9760-82, [1A4]) at 1:50 and 1:200, respectively in blocking buffer overnight at 4°C on a rocker. The tissue was rinsed 3 x 20 min with 0.1% Triton-X. The tissue was

then incubated with secondary antibody (goat anti-Armenian Hamster AI647 (Jackson ImmunoResearch Laboratories Inc., 127-605-160) at 1:500 in blocking buffer overnight at 4°C on the rocker. The samples were washed 3 x 20 mins with 0.1% Triton-X solution. Anterior segments were placed on a clean glass slide, facing up. The samples were mounted and a coverslip placed on top. The slides were left to dry overnight before sealing with nail polish and imaging. Images were taken using a Nikon Eclipse 90i confocal laser-scanning microscope (Melville). Z-stack images of 1 μm thickness were taken of each quadrant and converted into maximum projection images which were used for analysis. Vessel identity was established based on several criteria. Limbal venous vessels (distal to SC and to the limbal capillary plexus) were identified by connection to the SC via collector channel, by smooth muscle actin coverage, and by appearance of CD31 immunoreactivity. Limbal arterioles were distinguishable from limbal venules by the appearance of smooth muscle coverage, endothelial morphology (polarity of CD31 immunoreactivity showing alignment of endothelial cells parallel to longitudinal vessel profile), and a lack of collector channels. Limbal capillary loops were easily identifiable as they have small-caliber lumens, connect arterioles to venules, and were devoid of α-SMA immunoreactivity. Vessel diameters were measured from the CD31 channel with the vasometric plugin in ImageJ (FIJI) using a workflow improved from that described in (10). Briefly, vessels were traced and the surrounding image are cleared of background. A central through line was drawn along the length of the vessel using the segmented line tool and measuring cross lines, perpendicular to the through line, were automatically applied to measure vessel widths every five microns. Individual vessel measurements were averaged to give an average vessel width per whole mount image. All measurements were made on de-identified images by an observer unaware of genotype or treatment conditions.

Subconjunctival ocular injection

Mice were anesthetized systemically by intraperitoneal injection of ketamine (100 mg/kg) and xylazine (10 mg/kg). Mice were then placed on a heating pad. IOP measurements were initially taken using a handheld rebound tonometer. IOP measurements were then re-taken 10 min post anesthesia after IOP had bottomed

out due to anesthesia. 0.5% Proparacaine ophthalmic solution (Sandoz) was dropped onto each eye and left for 1-2 min. 10 µl of 50 µM 1400W (#81520, Cayman), an iNOS-selective inhibitor or 50 µM L-NAME (#81520, Cayman), a non-selective NOS inhibitor was injected into the subconjunctival region of one eye using a 30G needle. The contralateral control eye received a sham injection of PBS only. Once the conjunctival bleb decreased in size (10 min post injection), IOP measurements were taken again. Eyes were then quickly enucleated and placed in 4% PFA overnight at 4°C.

References

1. Lei Y, et al. Aqueous Humor Outflow Physiology in NOS3 Knockout Mice. *Investigative ophthalmology & visual science.* 2015;56(8):4891-8.
2. Gothert JR, et al. Genetically tagging endothelial cells in vivo: bone marrow-derived cells do not contribute to tumor endothelium. *Blood.* 2004;104(6):1769-77.
3. Burn SF. In: Michos O ed. *Kidney Development: Methods and Protocols*. Totowa, NJ: Humana Press; 2012:241-50.
4. Shimada A, et al. Improved methods for detection of β-galactosidase (lacZ) activity in hard tissue. *Histochem Cell Biol.* 2012;137(6):841-7.
5. Stamer WD, et al. eNOS, a pressure-dependent regulator of intraocular pressure. *Investigative ophthalmology & visual science.* 2011;52(13):9438-44.
6. Elliott MH, et al. Caveolin-1 modulates intraocular pressure: implications for caveolae mechanoprotection in glaucoma. *Scientific reports.* 2016;6:37127.
7. Sherwood JM, et al. Measurement of Outflow Facility Using iPerfusion. *PLoS One.* 2016;11(3):e0150694.
8. Reina-Torres E, et al. Aqueous Humor Outflow Requires Active Cellular Metabolism in Mice. *Investigative ophthalmology & visual science.* 2020;61(10):45-.
9. Li G, et al. Pilocarpine-induced dilation of Schlemm's canal and prevention of lumen collapse at elevated intraocular pressures in living mice visualized by OCT. *Investigative ophthalmology & visual science.* 2014;55(6):3737-46.
10. Li G, et al. Integral role for lysyl oxidase-like-1 in conventional outflow tissue function and behavior. *Faseb j.* 2020;34(8):10762-77.
